# Biomarkers common for inflammatory periodontal disease and depression: A systematic review

**DOI:** 10.1016/j.bbih.2022.100450

**Published:** 2022-03-14

**Authors:** Sudan Prasad Neupane, Anca Virtej, Lene Elisabeth Myhren, Vibeke Hervik Bull

**Affiliations:** a National Centre for Suicide Research and Prevention, Institute of Clinical Medicine, University of Oslo, Norway; b Oral Health Centre of Expertise in Rogaland, Stavanger, Norway

**Keywords:** Periodontitis, Depression, Neuroimmune, Biomarker, Systematic review

## Abstract

**Background:**

Dysregulated immune response arising in the periphery can induce depressive symptoms through neuroimmune interactions. Inflammatory oral pathology can be a potent inducer of chronic neuroimmune response relevant to depression. We aimed to synthesize available evidence for the association between inflammatory periodontal diseases (IPD) and major depression (MD) in relation to a broad range of biomarkers.

**Methods:**

Medline, Embase, PsycInfo, Cochrane Library, Web of Science and Scopus databases were searched from inception until January 27, 2022. Search terms included subject headings and synonyms for inflammatory periodontal disease and depression. Studies that reported data on both depression and inflammatory periodontal disease as categories along with measurement of a biomarker were considered. Two reviewers independently selected the articles for inclusion, extracted data and assessed the quality of each study. The protocol for this study was registered with PROSPERO, CRD42021215524.

**Results:**

Twenty-eight studies were included in the final review-eleven cross-sectional studies, seven case-control studies, and six prospective cohort studies conducted in humans; the remaining four were experimental animal studies. Eighteen studies including all animal studies reported a positive association between depression and periodontal disease; one study reported a negative association and another nine studies found no such associations. Twenty studies reported mixed associations between IPD and biomarkers (i.e, salivary, serum, urine or gingival crevicular fluid cortisol, C reactive protein, cytokines, etc.). Biomarkers related to depression were gingival crevicular fluid cortisol, interleukin 6 (IL-6), Il-1β, immunoglobulin G against Bacterioides forsythus; root canal lipopolysaccharides; blood IL-6, IL-1β, cortisol, advanced oxidation protein products, nitric oxide metabolites, lipid hydroperoxides and trapping antioxidant parameter; whereas five studies found no associations between depression and a biomarker. Although animal studies showed interaction of immune, inflammatory and neurotrophic biomarkers in the relationship between depression and periodontal disease, human studies showed mixed findings. In most studies, there were risks of bias due to the sample selection and assessment protocol. Study heterogeneity and limited number of comparable studies reporting on shared biomarkers precluded a meta-analysis.

**Conclusion:**

Immune-inflammatory contribution to depression was evident in the context of inflammatory periodontal diseases, but whether biomarkers mediate the associations between IPD and MD needs to be tested through methodologically rigorous studies aiming specifically at this hypothesis.

## Highlights

• Inflammatory signals from the periphery induces brain neuroimmune response.
• Periodontal inflammation is a potential source of neuroinflammation in depression.
• We did a systematic review on biomarkers shared by periodontitis and depression.
• Biomarkers relevant to immune changes in depression was also found in periodontitis.
• Studies should directly test biomarker roles in periodontitis–depression relations.

## 1 Introduction

For a long time, depression and periodontitis remained appraised as diseases localized within the cranial and oral cavities, respectively. However, recent findings suggest that pathological processes involved in these conditions have far-reaching consequences. In particular, the field of psychoneuroimmunology has established that inflammatory signals arising in the periphery can reach brain areas responsible for mood and behavioral regulation (Raison et al., 2006). In this context, oral pathology including periodontal diseases can be a putative source of neuroimmune dysregulation observed in depressive illness (Hashioka et al., 2019). The evidence base for this hypothesis remains to be established.

Considerable amount of data suggests an increased occurrence of mental health issues in persons with inflammatory oral diseases, and vice versa (Aldosari et al., 2020; Choi et al., 2021; Zheng et al., 2021). A 12-year follow up study of adults (N ​= ​12,708) with newly diagnosed periodontitis showed a 73% increased risk of depression during the follow-up period regardless of sex, age and other comorbidities at baseline (Hsu et al., 2015). Most studies included in a systematic review (Peruzzo et al., 2007) showed a positive relationship between stress/psychological factors and periodontal diseases. Furthermore, associations between psychological stress and periodontitis are likely related to or potentially mediated through salivary and blood cortisol, lipopolysaccharides and other markers of cellular and systemic stress and inflammation (Gomes et al., 2018; Goyal et al., 2011; Hilgert et al., 2006; Ishisaka et al., 2008). Stress causes immune responses that increase susceptibility to infection and potentially contribute to the progression of periodontitis (Warren et al., 2014). In an animal model of depression, periodontal ligature-induced bone loss was greatly increased while exhibiting psychological, behavioural and neurochemical responses consistent with depression (Breivik et al., 2006). Together, this evidence indicates bidirectional causal pathways for the comorbidity between depression and periodontal disease and for shared inflammatory mediators.

Altered levels of immune-inflammatory markers including pro-inflammatory cytokines, oxidative and nitrosative stress markers, neurotoxic metabolites of tryptophan degradation and reduced neurotrophic levels are detected in a considerable proportion of individuals with depression (Mac Giollabhui et al., 2021; Maes et al., 2011; Mariani et al., 2021; Osimo et al., 2019; Raison and Miller, 2013). A recent meta-analysis of 82 studies showed that levels of the cytokines Interleukin 6 (IL-6), tumor necrosis factor alpha (TNF-α), IL-10, the soluble IL-2 receptor, C–C motif chemokine ligand 2 (CCL2), IL-13, IL-18, IL-12, the IL-1 receptor antagonist, and the soluble TNF receptor 2 were elevated in patients with depression compared to healthy control subjects, whereas interferon gamma (IFN-γ) levels were lower in depression (Köhler et al., 2017). These broad alterations of immune signaling molecules have been proposed as being both cause and effect of depression (Raison and Miller, 2011). The proposed sources of inflammation in depression have been summarized as psychosocial stressors, poor diet, physical inactivity, obesity, smoking, altered gut permeability, atopy, dental caries, sleep and vitamin D deficiency (Berk et al., 2013). For these proximal causes, research literature has focused mainly on local or regional inflammatory flux but distal consequences of the tightly regulated immune and inflammatory responses beyond the primary sites remain to be delineated. Thus, there is a need to identify novel biomarkers that are likely to be reliable in predicting the depressive comorbidity in periodontitis, while also being easily implementable in clinical practice.

**Fig. 1:**
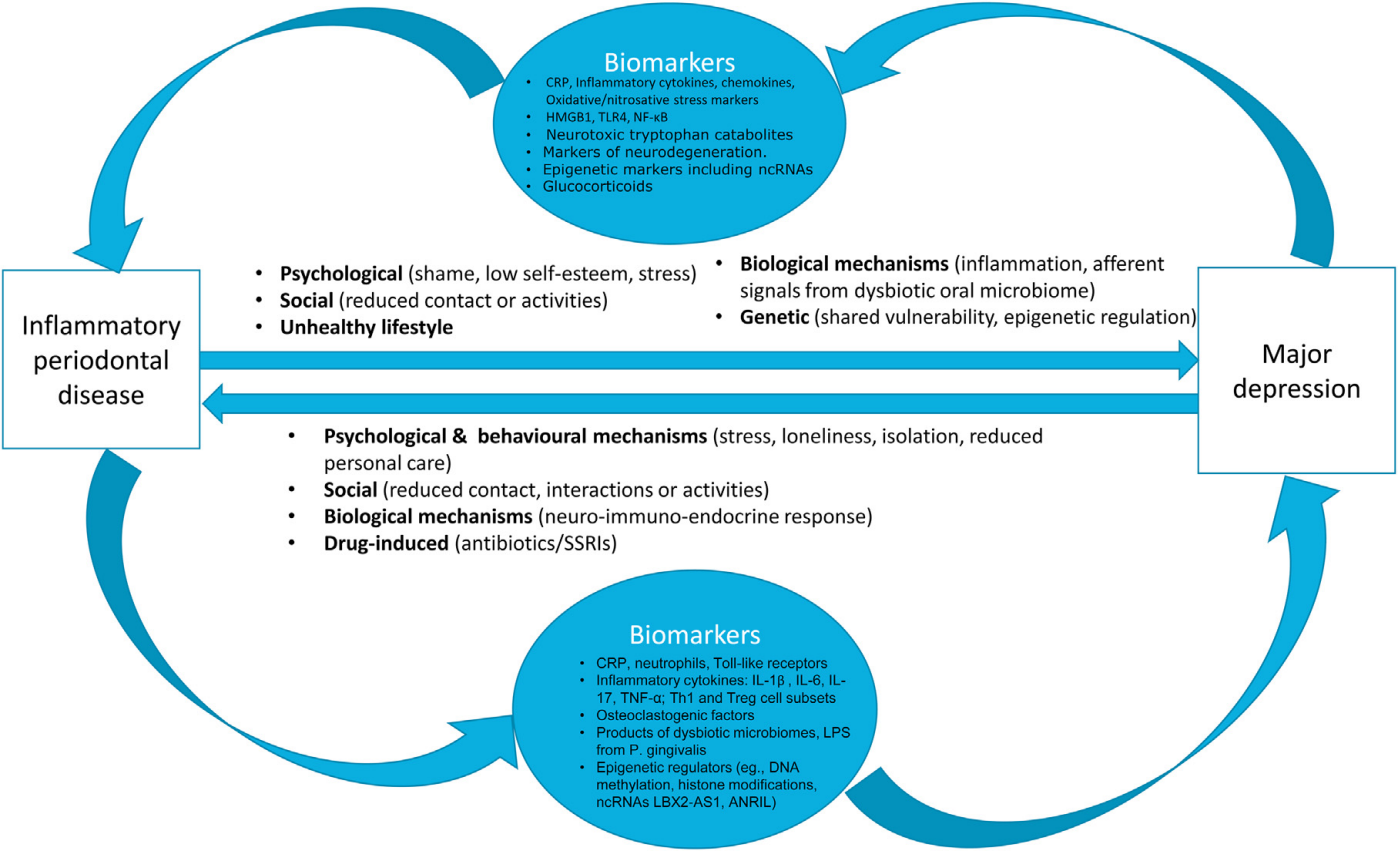
Schematic showing biomarkers as putative connecting links between inflammatory periodontal disease and depression. Comorbidity between depression and inflammatory periodontal diseases can be explained in terms of psychological, social, pharmacological and biological, including genetic factors. The bidirectional associations are potentially mediated by a range of inflammatory biomarkers such as cytokines, chemokines, cytotoxic products of metabolic pathways, stress biomarkers, products of oral/gut microbiome, osteoclastogenic factors and epigenetic regulators. Factors responsible for inciting periodontal and depression pathologies may originate from oral microbes, periostial tissue as well as the central nervous system and systemic circulation. CRP: C-reactive protein, HMGB1: high mobility group box 1, IL: interleukin, TNF-α: tumor necrosis factor alpha, TLR: Toll-like receptor, NF-κB: nuclear factor kappa-light-chain-enhancer of activated B cells, Th1 cells: T Helper 1 Cells, Treg: Regulatory T cells, LPS: lipopolysaccharide, ncRNA: non-codingRNA, LBX2-AS1: LBX2 Antisense RNA 1, ANRIL: Antisense Noncoding RNA in the INK4 Locus.

There are two lines of evidence that suggest inflammatory periodontal diseases (IPD) as a plausible source of such inflammation in depression. Firstly, IPD involves dysbiosis of the oral microbial community in favor of inflammation-provoking pathogens (predominantly *Porphyromonas gingivalis*) through release of bacterial proteins such as lipopolysaccharides that not only activate systemic low-grade inflammation but also downregulate neurotrophic factor maturation in the brain (Wang et al., 2019). Secondly, overzealous host immune activation in direct response to local tissue destruction in IPD leads to exaggerated osteoclastic activity and immune dysregulation (Pan et al., 2019; Zaric et al., 2010). These signals can propagate through multiple humoral, cellular and neural routes (Maier and Watkins, 1998; Quan and Banks, 2007; Watkins et al., 1995), ultimately reaching the brain whereby neuroinflammation is set in-a finding frequently reported in patients with depression. Thus, stress-mediated efferent neuroendocrine and immune signaling in depression as well as afferent inflammatory signals arising from IPD point towards a compelling biological pathomechanism for bidirectional associations between depression and IPD (see Fig. 1). It is of great relevance to study whether empirical evidence supports such mechanistic explanations for these frequently co-occurring and burdensome disorders of great societal significance. We are not aware of any other research projects that directly test this plausible but novel hypothesis. Thus, the objective of this study is to summarize the evidence on the association between inflammatory periodontal diseases and depression mediated by or related to a broad range of biomarkers that are detectable in circulatory and local fluids.

## 2 Methods

### 2.1 Search strategy and selection criteria

We conducted a systematic review and attempted meta-analysis following the Preferred Reporting Items for Systematic Reviews and Meta-Analyses (PRISMA) (Moher et al., 2015). Records were searched in the databases Medline (Ovid), Embase (Ovid), PsycInfo (Ovid), Cochrane Library, Web of Science and Scopus from database inception to 27. Jan 2022. Search strings included a wide range of subject headings and synonyms for inflammatory periodontal disease (such as gingivitis, necrotizing ulcerative gingivitis, periimplantitis, periodontitis or periodontal pocket, pericoronitis or gingival hemorrhage) and depression (such as depression or depressive disorder). Full details of the search strategy are available in the *Supplementary material (*Appendix 1.0-1.1*)*. Two authors screened the title and abstract of 1557 reports independently using Rayyan-a web-based screening solution (Ouzzani et al., 2016).

**Fig. 2:**
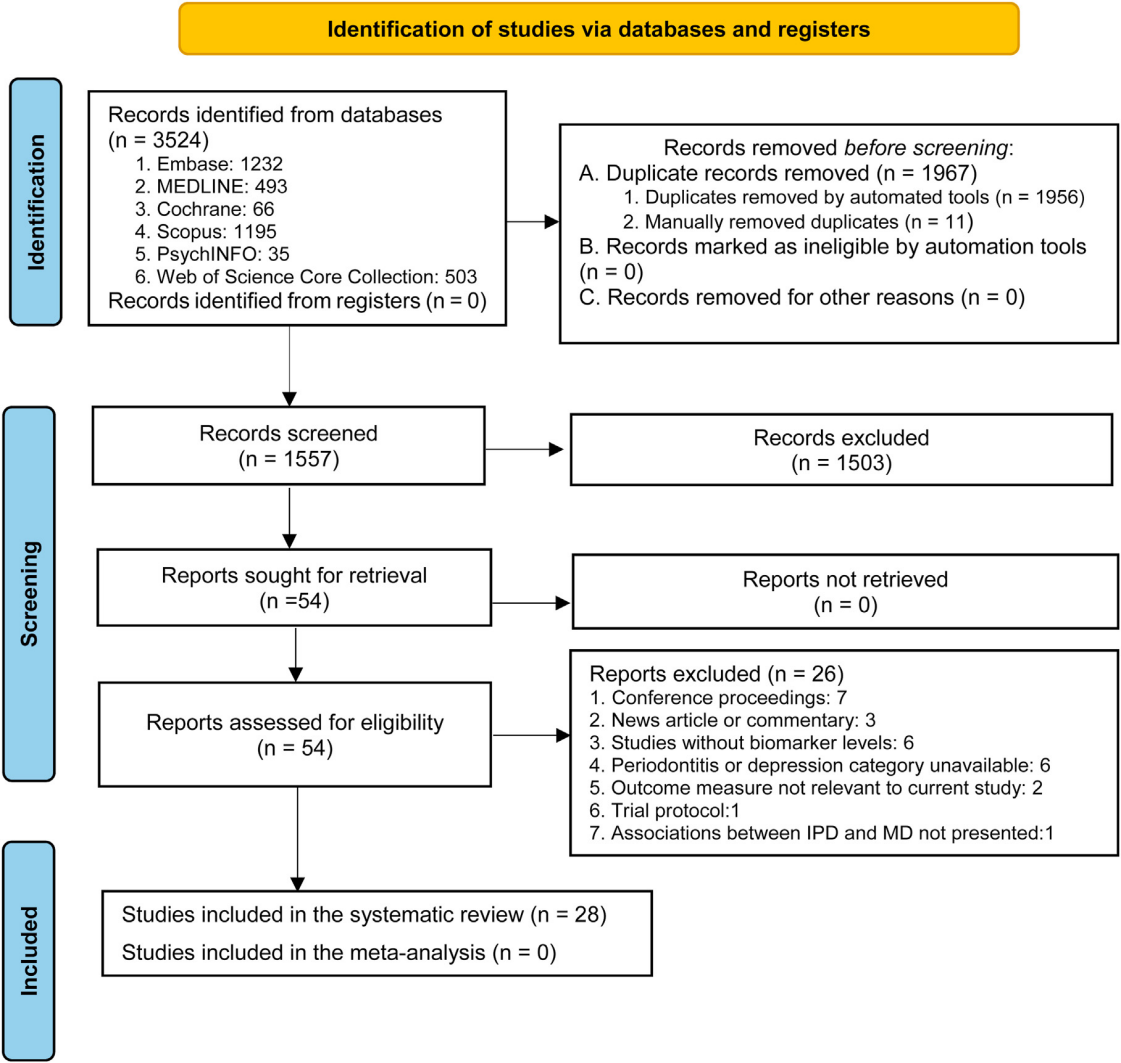
Preferred Reporting Items for Systematic Reviews and Meta-analysis flow diagram for the current study.

Inclusion criteria: (a) original peer-reviewed studies conducted in vivo, in humans or animal models, (b) either sex, (c) any age, (d) any human or animal samples, (e) examined both inflammatory periodontal disease and depression, and (e) reported on at least one biomarker. A broad definition of biomarkers was used as suggested by the US National Institutes of Health (National Institutes of Health, 2001). Exclusion criteria: (a) qualitative studies, (b) case reports, (c) opinion articles/letters to the editor, (d) conference proceedings/reviews, (e) studies without biomarker levels analysis, (f) studies where average values and spread of the biomarker levels at group or individual levels not presented or unavailable after contacting the corresponding author, (g) full text not accessible, and (h) articles published in languages other than English or a Scandinavian language. For cases where reports were evaluated differently between two authors, a third author was invited to resolve. Fifty-four articles that screened positive were full text reviewed by two authors. If relevant data were missing from a report, the corresponding authors were contacted and were requested for additional information. We excluded 26 reports due to violation of inclusion criteria (Fig. 2), leaving 28 reports. The review was registered on PROSPERO (CRD42021215524) before the systematic review was done.

### 2.2 Data extraction and analysis

Data were independently extracted by all authors in pairs using a standardized form. A third reviewer crosschecked the extraction sheet for each study for consistency and any discordance was resolved through discussion among the authors.

We included studies under an umbrella of depression for any categories the authors defined as satisfying depression diagnosis or having adequate symptom load for depression. We operationalized IPD with any criteria for periodontal inflammation presented by the authors, but included studies that reported values of at least one of the three parameters, i.e, bleeding on probing (BOP), pocket probing depth (PPD) and clinical attachment level (CAL) necessary for a periodontal diagnosis.

We closely evaluated the data against study design, study population and settings for possible meta-analysis of biomarkers reported in comparable studies. As a priori, at least three methodologically comparable studies reporting the same biomarkers would provide basis for further meta-analysis.

### 2.3 Quality assessment

Key confounding variables considered in each study are presented in Supplementary material (Appendix 2). Potential risks of bias in animal studies were assessed using the SYRCLE's RoB tool (Hooijmans et al., 2014), an adapted version of the Cochrane RoB tool. Observational cohort, cross-sectional and case-control studies on humans were assessed using the Quality Assessment Tools of the National Heart, Lung, and Blood Institute. The guidelines are available through https://www.nhlbi.nih.gov/health-topics/study-quality-assessment-tools, and the tools contain 14 criteria (cohort and cross-sectional) or 12 criteria (case-control studies) assessing potential selection, information and measurement biases or confounding factors, rated as yes, no, not reported, cannot determine, or not applicable. One point was given for yes, and 0 point for the other responses. Two authors graded each study independently, and the grading was subsequently discussed to reach consensus. Risk of bias matrices are presented as supplementary material (Appendix 3a,3b,3c).

## 3 Results

### 3.1 General study characteristics

Of the 28 studies included in the present systematic review, eleven were cross-sectional studies, seven were case-control studies, and six were prospective cohort studies conducted in humans. Remaining four studies were conducted in animals. Human studies were mostly based on dental in- or outpatients and the sample size varied between 30 and 600 individuals. Table 1 Shows detailed information about the study design, population characteristics, sample size, periodontal and depression status, diagnostic criteria as well as the association between periodontal disease and depression.

**Table 1:** Individual study details along with operationalization for depression and inflammatory periodontal diseases, associations between the two and study quality.

Author (year), country	Sample size	Population, mean age (SD/range), % female	Comparison groups	Inflammatory periodontal disease diagnosis/criteria	Depression diagnosis/criteria	Direction of IPD-MD association	Association between IPD and MD	Study Quality
**Animal studies**								
Breivik et al. (2006), Norway	40	Rats, 13 weeks, 0% female	Animals with olfactory bulbectomy vs sham operated	Ligature-induced periodontitis (alveolar bone loss)	Experimental (depression-like behavior induced by olfactory bulbectomy)	+ve	Periodontal bone loss was elevated (1.06 ​± ​0.25 ​mm) in depressed rats vs (0.90 ​± ​0.13 ​mm) sham-operated control rats (p ​< ​0.01), and reversed with tianeptine.	See appendix 3a.
Breivik et al. (2015), Norway	43: 12 controls; 31 cases	Rats, 13 weeks, 56% female	Animals with maternal deprivation vs no maternal deprivation	Ligature-induced periodontitis (alveolar bone loss)	Maternal deprivation- induced anxiety and depression-like behavior	+ve	Significantly more severe periodontitis in depressed rats.	See appendix 3a.
Martínez et al. (2021), Spain	47: 11 controls; 12 IPD; 12 MD; 12 IPD ​+ ​MD	Rats, 0% female	Animals with induced periodontitis or depressive-like behavior alone or in combination	Rat model of periodontitis induced by oral inoculation of *P. gingivalis* and *F. nucleatum*	Experimental depression model using chronic mild stress	+ve	Rats with IPD and depressive-like behavior had significantly higher GI values compared to non-depressed rats. In addition, IPD ​+ ​MD rats had more alveolar bone loss	See appendix 3a.
Wang et al. (2019), China	18	Mice, 6 weeks, 100% female	Animals induced with periodontitis vs those receiving vehicle	Mouse model of periodontitis; (alveolar bone loss) induced by oral inoculation of *P. gingivalis*	Behavioral mouse model of depression (forced swim and tail suspension tests)	+ve	Periodontal disease induced by *P. gingivalis* or LPS caused depression-like behavior in mice.	See appendix 3a.
Animal studies: Total studies with positive associations between IPD and MD: 4; negative associations: 0; not shown or unclear: 0								


***Cross-sectional studies (Human)***								
Cakmak et al. (2014), Turkey	120: 40 control subjects; 41 localized periodontitis; 39 generalized periodontitis	Dental outpatients, 38.3 (24–63) yrs, 51% women	Patients with chronic localized or generalized periodontitis vs healthy controls	Chronic Periodontitis – localized (patients with <7 sites having PD ​≥ ​5 ​mm and CAL≥4 ​mm) or generalized (patients with ≥7 sites having PD ​≥ ​5 ​mm and CAL≥4 ​mm).	Major depression (BDI- Beck Depression Inventory)	–	No difference in depression scores between IPD groups	fair
Cakmak et al. (2016), Turkey	92: 31 control subjects; 61 periodontitis	Dental outpatients, 24–60 ​yrs, 49% women	Patients with chronic periodontitis and aggressive periodontitis vs healthy controls	Generalized chronic periodontitis (PD ​≥ ​5 ​mm; CAL≥5 ​mm at all quadrants and BOP in ≥50% of the sites. Generalized aggressive periodontitis (severe alveolar bone loss and PPD≥6 ​mm and CAL≥6 ​mm on ≥8 teeth)	Major depression (BDI- Beck Depression Inventory)	+ve	Depression scores significantly elevated in the aggressive periodontitis group, but not in the chronic periodontitis group.	good
da Silva et al. (2015), Brazil	64: 21 healthy controls; 43 patients with gingivitis	School students, 11.4 (11–12) yrs., 67% women	Patients with gingivitis vs healthy controls	Gingivitis (based on interview, BOP, Community periodontal index with bleeding on six index teeth)	Depression (CDI- Children's depression inventory)	–	No correlation between MD and IPD. Mean depression score in gingivitis 12.3 (SD 9.1) vs no gingivitis 10.0 (SD 8.6).	fair
Fenol et al. (2017), India	70	Prison inmates, 38.6 ​± ​10.9 (25–60) yrs., 0% women	Patients with moderate and severe periodontitis vs healthy controls	Periodontitis Group A (PPD≥4 and ˂6 ​mm), Group B (PPD≥6 ​mm in at least 4 sites) and controls (PPD≤3 ​mm)	Depression (DASS- Depression, anxiety and Stress Scale)	+ve	Significant associations between high stress levels (including depression symptom load) and periodontitis measures.	fair
Gomes et al. (2018), Brazil	47	Dental outpatients, ≈43.5 (18+) yrs., 60% women	Patients with chronic apical periodontitis with and without depression	Chronic apical periodontitis (based on clinical and radiographic exam)	Major depression (Beck Depression Inventory and Hamilton Depression Rating Scale)	+ve	Higher depression scores in chronic apical periodontitis group (BDI 13.5 ​± ​1.9 vs 2.3 ​± ​3.1; HDRS 11.5 ​± ​1.0 vs 3.6 ​± ​1.7	fair
Katuri et al. (2016), India	70: 23 with depression; 24 no depression; 23 yoga-practitioners	Dental outpatients, 46.83–47.13 ​yrs, 53% women	All patients with chronic periodontitis divided in 3 groups: with/without depression and yoga-practicing	Chronic periodontitis (based on PI, PPD 5–8 ​mm, CAL>5–8 ​mm)	Depression (ZSDS-Zung's Self-rating Depression Scale)	–	No difference between the groups with regards to periodontal parameters.	poor
Rahate et al. (2021), India	90: 30 healthy controls; 30 periodontitis; 30 periodon titis/smokers	Dental outpatients, 49–52 ​yrs, 38% women	Comparison between periodontally healthy patients and stage III periodontitis patients with or without a history of smoking.	Stage III periodontitis (based on PPD ≥6 ​mm, CAL ≥5 ​mm, radiographic bone loss extending beyond middle third of the root	Mild, moderate or severe depression (Zung's Self-rating Depression Scale)	+ve	Patients with periodontitis reported increased depression compared to controls.	fair
Refulio et al. (2013), Peru	70: 36 with periodontitis; 34 healthy controls	Dental outpatients, 30–65 ​yrs, 64% women	Patients with periodontitis vs healthy controls	Chronic periodontitis (>3 sites ​+ ​probing pocket depth >4 ​mm). Localized (<30% of teeth affected) or generalized (>30% of teeth affected).	Depression (Zung's Self-rating Depression Scale)	+ve	All patients with chronic periodontitis received depression diagnosis.	poor
Rodriguez Franco et al. (2020), Mexico	61: 35 periodontitis; 10 MD; 16 depressive symptoms	Psychiatric inpatients, 46.4 ​yrs, 51% women	Patients with periodontitis vs patients with depression	Periodontitis (based on BOP, CAL, PD).	Depressive symptoms (BDI) and major depression	-ve	Depression negatively predicted clinical attachment loss in their model.	fair
Rosania et al. (2009), USA	45	Dental outpatients, 45–82 ​yrs, 69% women	Recall patients with periodontitis vs patients with periodontitis and depression	Periodontal disease (based on CAL, recession, PPD with severity grades)	Self-reported depression (CES-D)	+ve	Positive correlations between missing teeth and depression (r ​= ​0.54, P ​< ​0.001)	fair
Solis et al. (2016), Brazil	72: 36 MDpatients; 36 healthy controls	Psychiatric outpatients, 18–58 ​yrs, 82% women	Patients with depression vs healthy controls	Periodontal examination (PI, GI, CAL, PD, missing teeth, previously reported in Solis et al. (2014))	Major depressive disorder (Structured Clinical Interview for DSM-IV), HAM-D-31)	–	Periodontal clinical parameters were indifferent between patients with and without depression.	good
Cross-sectional studies (human): Total studies with positive associations between IPD and MD: 6; negative associations: 1; not shown or unclear: 4								


***Case-control studies (Human)***								
Bawankar et al. (2018), India	75: 25 controls; 50 IPD patients	Dental outpatients, 30–65 ​yrs, 70% women	Healthy controls vs smokers with IPD and non-smokers with IPD	Chronic untreated severe periodontitis (based on CAL ≥5 ​mm, PPD≥5 ​mm, >30% of teeth affected evidence of radiographic bone loss.	Depression (Zung's self-rating depression score SDS)	+ve	Mean depression score significantly higher in IPD than controls.	fair
Cohen-Cole et al. (1983), USA	70: 35 healthy controls; 35 patients with gingivitis	Dental outpatients ≈24.3 (14–33) yrs., 60% women	Patients with gingivitis vs healthy controls	Acute necrotizing ulcerative gingivitis (ANUG, Trench Mouth) (based on pain and interdental papillae necrosis)	Depression (Center for Epidemiologic Studies Depression Scale)	+ve	Elevated depression score in ANUG patients. OR for MD in IPD ​= ​4.24	fair
Johannsen et al. (2006), Sweden	72: 43 MD patients and 29 controls	42 ​± ​9.3 ​yrs (patients); 54.5 ​± ​2.9 ​yrs (controls), 100% women on long term sick leave	Patients with depression vs healthy controls	Clinical periodontal examination (dental plaque, GI, BOP, PPD, CAL, tooth number). Specific criteria not reported.	Major depression (Structured Clinical Interview for DSM-IV)	+ve	MD patients had significantly higher amount of dental plaque (0.18 ​± ​0.13 vs. 0.10 ​± ​0.10 unit) and higher GI (1.53 ​± ​0.26 vs. 0.89 ​± ​0.35) than control subjects.	fair
Johannsen et al. (2007), Sweden	49: 29 controls, 20 MD patients	≈52.1 ​± ​4.5 ​yrs, 100% women on long term sick leave	Patients with depression vs healthy controls	Gingivitis (based on CAL, BOP, inflammation, plaque, PPD).	Major depression (Structured Clinical Interview for DSM-IV)	+ve	MD patients had significantly worse periodontal parameters; dental plaque including gingival inflammation, but not increased clinical attachment level.	fair
Karimi et al. (2017), Iran	30: 15 with periodontal disease; 15 healthy controls	Dental outpatient, 42.4 ​± ​5.4 ​yrs (cases); 44.5 ​± ​8.4 ​yrs (controls), 40% women		Periodontal disease (BOP, CAL, PPD, GI, PI)	Self –reported depression (DASS42)	+ve	87% with periodontal disease had depression compared with 60% without periodontal disease	poor
Leira et al. (2019), Spain	179: 102 with migraine; 77 healthy controls	Dental outpatients, 47 ​yrs, ≈97.7% women	Patients with migraine vs non-migraine	Mild, moderate or severe periodontitis (based on PPD, CAL and surface area of bleeding pocket).	Depression (undefined)	–	IPD correlated with migraine that again was highly correlated with depression; however direct associations were not reported.	fair
Moss et al. (1996), USA	148: 71 patients; 77 controls	Dental outpatients, 44.4 (8.6) yrs. (case); 43.8 (9.3) yrs. (controls), % women unknown	Patients with periodontal disease vs healthy controls	Periodontitis (based on PI, BOP, CAL and PPD)	Depression (Brief symptom inventory)	+ve	Depression was associated with extensive periodontal disease. Odds ratio for depression among periodontitis vs controls 1.28 (95% CI 0.56–2.95).	fair
Case-control studies (Human): Total studies with positive associations between IPD and MD: 6; negative associations: 0; not shown or unclear: 1								


***Prospective cohort studies (Human)***								
Cakmak et al. (2019), Turkey	55:15 healthy controls; 40 subjects with periodontitis	Dental outpatients, 40.4 (26–63) yrs., 40% women	Patients with chronic localized and generalized periodontitis vs healthy controls	Localized or generalized chronic periodontitis (based on PI, GI, BOP, PPD, CAL). Localized: PPD ≥4 ​mm and CAL ≥3 ​mm at ≥2 sites and BOP in ≥30% sites Generalized: PPD ≥5 ​mm and CAL ≥6 ​mm at multiple sites and BOP in ≥60% sites.	Major depression (BDI- Beck Depression Inventory)	–	No difference in depression scores after IPD treatment.	good
Kurer et al. (1995), UK	47	Dental outpatients, 20–50 (not specified), gender distribution not reported	All patients with high level of oral health	Gingivitis (based on Loe & Silness gingival index, modified Quigley and Hein plaque index, no PPD>5 ​mm).	Depression/anxiety/psychological mood	–	Depression was associated with plaque level (r ​= ​0.28; p ​< ​0.05), but not gingivitis.	fair
Nascimento et al. (2019), Brazil	539	Population-based, 31 ​yrs, 49% women	Prevalence of periodontitis and depression	Periodontitis (based on CAL and BOP).	Major depressive episode (MINI) and depressive symptoms (BDI)	+ve	Higher risk of periodontitis (RR 1.19) and more severe periodontitis in patients with depressive symptoms but not diagnosis.	fair
Petit et al. (2020), France	71	Dental inpatients, 51.3 ​yrs, 30–40% women	Patients with depression	Severe generalized chronic periodontitis (based on PI, BOP, PD, CAL with at least 5% of the sites withPD ​> ​5 ​mm and bone loss).	Self-reported depression (DASS42)	+ve	Increased stress levels associated positively with worsened outcomes after non-surgical periodontal treatment.	good
Petit et al. (2021), France	71	Dental outpatients, 51 ​± ​10 ​yrs, 56% women	Periodontitis patients with depression	Severe generalized chronic periodontitis (based on 5% of sites with PD ​> ​5 ​mm and radiographic evidence of bone loss).	Self-reported depression (DASS42)	–	19% of patients suffered from depression. Increased depression resulted in worsened outcomes after non-surgical periodontal treatment	Good
Zhang et al. (2021), China	600: 200 healthy controls, 200 IPD only; 200 IPD+MD	Dental outpatients, 20–50 ​yrs, 50% women	Healthy controls versus periodontitis patients with (+smoking) or without depression (-smoking).	Chronic periodontitis (based on PPD ≥5 ​mm, CAL ≥5 ​mm on more than 30% of teeth and radiographic evidence of bone loss).	Self-reported depression (SCL-90)	–	Depression in chronic periodontitis was associated with higher periodontal destruction, potentially mediated through smoking.	fair
Prospective cohort studies (Human): Total studies with positive associations between IPD and MD: 2; negative associations: 0; not shown or unclear: 4								

Notes: IPD: inflammatory periodontal disease, MD: major depression, BDI: Beck Depression Inventory, HDRS: Hamilton Depression Rating Scale, DASS: Depression, Anxiety and Stress Scale, CAL: clinical attachment level, PPD: probing pocket depth, GCF: gingival crevicular fluid.

### 3.2 Association between IPD and MD

All four animal studies included in this review reported a positive association between depression-like behavior and periodontal disease. Among human studies, fourteen studies found a positive association between periodontal disease and depressive disorder or depressive symptoms suggestive of MD diagnosis. Cakmak et al. (2016) reported elevated depression scores in patients with aggressive periodontitis but not in patients presenting with chronic periodontitis. Nascimento et al. (2019) found higher risk of periodontitis in patients with depressive symptoms but not among those with depression diagnosis, while Gomes et al. (2018) reported higher depression scores among patients with chronic apical periodontitis. Fenol et al. (2017) found significant associations between high levels of stress (including depression) and worse periodontal status. Finally, depression in chronic periodontitis was associated with higher periodontal destruction (Zhang et al., 2021) and stress levels indicative of depression were found to be associated with poorer periodontal healing among IPD patients (Petit et al., 2020, 2021).

Nine of the included studies found no associations between depression and periodontal disease. Yet another study (Johannsen et al., 2006) did find an association between depression and dental plaque and gingival inflammation but did not observe any significant associations for other clinical periodontal parameters (BOP, PPD, CAL, number of teeth).

Only one study (Rodriguez Franco et al., 2020) found depression to be negatively associated with periodontal disease.

### 3.3 Biomarkers

Details about measured biomarkers, sample source and assay method are presented in Table 2. The studies varied greatly between biomarkers that were investigated, type of sample that was used and assay method. The biomarkers reported in the studies generally fell into one or more of the following categories: markers of psychological stress, oxidative and nitrosative stress and damage, bacteria or endotoxin, immunoglobulins, hormones or their receptors, neurotrophic factors and inflammatory cytokines. Cortisol was measured in the majority of the studies, either in saliva, serum or gingival crevicular fluid.

**Table 2:** Biomarkers reported in each study by depression and inflammatory periodontal disease, and the role of biomarker in the relationship between the two conditions.

Study	Biomarker	Sample source and assay method	Results: Biomarker by depression	Results: Biomarker by IPD	Role of biomarker in the relationship between IPD and MD
**Animal studies**					
Breivik et al. (2006)	Cortisol, TNF-α, TGF-1β, IL-10, GR expression	Serum, hippocampal tissue/RIA (radioimmunoassay) for cortisol; ELISA for TGF-β; qRT-PCR for GR mRNA	Decreased GR expression in hippocampus of depression model rats.		IPD ​+ ​MD rats had significantly higher (1140 ​± ​388 ​ng/ml) serum cortisol levels compared to IPD only rats (756 ​± ​423 ​ng/ml). IPD ​+ ​MD rats also had higher TGF-1β (16 ​± ​4 vs. 12 ​± ​3) and decreased TNF-α (562 ​± ​644 vs. 2450 ​± ​2506) levels. Upon LPS stimulation, compared to controls (627 ​± ​569 ​ng/ml), significantly higher serum cortisol levels were found in the depression model rats (1017 ​± ​606 ​nm/L), demonstrating that the bulbectomy induced a stronger HPA axis responsiveness to the inflammatory LPS. Depression induced hyper- responsiveness of HPA axis (indicated by cortisol level) was not amenable to antidepressant treatment although TGF-1β and TNF-α changes were reversed.
Breivik et al. (2015)	Cortisol, TGF-1β, IL-10, TNF-α; GR expression	Serum, hippocampal tissue/RIA (radioimmunoassay) for cortisol; ELISA for TGF-1β; qRT-PCR for GR mRNA	Decreased GR expression in hippocampus of depression model rats.		Depression models of rat on top of ligature-induced IPD had higher hippocampal GR Expression, and lower serum TGF-1β levels after LPS stimulation. TGF-β levels (pg/ml) in depression group (male 56 ​± ​3; female 51 ​± ​5) vs. healthy control group (male: 63 ​± ​7, female 58 ​± ​7); However, cortisol, IL-10 and TNF-α levels did not differ by depression status.
Martínez et al. (2021)	Corticosterone, GR receptor, LPS and 16S rRNA genes of *P. gingivalis*, *F. nucleatum,* LPS binding protein, TNF-α, IL-1β, NF-kB (p65 subunit), TLR-4, iNOS, mPGES, phosphor p38 MAPKa/b subunit, APO -A1 phospho-mTOR/mTOR ratio	Plasma, frontal cortex (nuclear extract or homogenate)/ELISA, RT-PCR, Western blot	Rats with depression like behavior had significantly upregulated expression of pro-inflammatory mediators (TNF-α, IL-1β, TRL-4, iNOS and p-p38) in the brain compared to controls.	Rats with IPD had significantly upregulated mRNA expression of TNF-α, and microsomal prostaglandin E synthase (mPGES) compared to controls.	Rats with IPD and depression-like behavior had increased expression of pro-inflammatory markers (TNF-α, IL-1β) in the brain. In addition, *F. nucelatum* was found in the brain parenchyma. These rats also had increased levels of plasma corticosterone and expression of glucocorticoid brain receptors.
Wang et al. (2019)	Cortisol, p75NTR, BDNF, TNF-α, IL-6, IL-1α	Serum, hippocampus, astrocytes, blood,	Depression-like behavior in periodontitis mice models induced with *P. gingivalis* had increased number of activated astrocyte and reduced levels of mature BDNF. These effects were reversed by TLR-4 inhibitor TAK242.	*P. gingivalis* inoculation and LPS from *P. gingivalis* caused increased alveolar bone loss (mandible) in mice. The mice had significantly elevated serum IL-1α and TNF-α and cortisol levels as well as PFC and hippocampal TNF-α, IL-6 and IL-1a expression compared with the control group.	Periodontal mouse model showed downregulated BDNF maturation through astrocytic p75NTR leading to depression like behavior.

***Cross-sectional studies (Human)***					
Cakmak et al. (2014)	Cortisol, DHEA	GCF/ELISA		Higher DHEA (pg/ml) levels in local (64 ​± ​31) as well as generalized chronic periodontitis (78 ​± ​39) compared to patient controls without IPD (59 ​± ​23). Cortisol levels did not differ across IPD groups.	
Cakmak et al. (2016)	Cortisol, DHEA, Salivary flow rate	GCF, Saliva/ELISA		GCF cortisol, saliva cortisol, GCF DHEA and saliva DHEA are elevated, both in generalized and localized chronic periodontitis groups compared to periodontally healthy patients.	
da Silva et al. (2015)	Cortisol	Saliva/Enzyme immunoassay		No significant differences in the diurnal decline of salivary cortisol between IPD patients and controls (0.17 ​± ​0.09 vs. 0.24 ​± ​0.21 ug/dl).	There was a strong correlation between MD and biomarker (diurnal decline in salivary cortisol) in IPD group (r ​= ​−0.64; p ​< ​0.01), but not in control group (r ​= ​0.07, NS).
Fenol et al. (2017)	Cortisol	Saliva/RP Elecsys kit	No significant association between salivary cortisol and depression level	Higher salivary cortisol 26 ​± ​4 (severe IPD), 19 ​± ​2 (moderate IPD) vs. control group 9 ​± ​3 units; P ​= ​0.001). Salivary cortisol correlated positively and significantly with CAL and PP	
Gomes et al. (2018)	LPS, LOOH, Nox, TRAP, AOPP, PON1	Root canal tissue (LPS), plasma/ELISA	There were significant correlations between root canal LPS and depression measured with the HDRS (r ​= ​0.8, p ​< ​0.001, n ​= ​47) as well as the BDI scale (r ​= ​0.8, p ​< ​0.001, n ​= ​47).	Clinical depression was significantly associated with increased root canal LPS, plasma AOPP, NOx, LOOH, and TRAP values, while there were no significant effects of –SH groups and PON1 activity.	Patients with IPD ​+ ​MD had greatly increased root canal LPS level as compared to IPD-MD group. In subjects with IPD, there were significant correlations between root canal LPS and HDRS (r ​= ​0.7, p ​< ​0.001, n ​= ​34) and the BDI (r ​= ​0.7, p ​< ​0.001, n ​= ​34). Association between depression and IPD was attributable, at least in part, to increased root canal LPS levels in IPD patients.
Katuri et al. (2016)	Cortisol	Serum	Serum cortisol levels and depression scores were higher in periodontitis patients with stress vs. with those without stress/yoga practitioners		
Rahate et al. (2021)	Cortisol, Ghrelin	Serum, saliva/ELISA	There was a positive correlation between salivary cortisol and depression scores in IPD patients (coefficient r ​= ​0.45 (non-smokers) and 0.40 (smokers)). Ghrelin levels were decreased in depressed IPD patients compared to IPD-only patients.	Cortisol levels were significantly elevated in IPD patients compared to controls in serum (16.4 ​± ​8.9 vs. 11.6 ​± ​5.5 pg/mL; P ​< ​0.0001) and saliva (399.7 ​± ​107 vs. 22.55 ​± ​7.0 ​pg/mL; P ​< ​0.0001). Ghrelin levels were elevated in IPD patients in both serum (650.25 ​± ​260.86 vs. 547.6 ​± ​166.5 ​pg/mL; P ​> ​0.0001) and saliva (892.4 ​± ​271.7 vs. 787.3 ​± ​230.3 ​pg/mL; P ​> ​0.0001).	
Refulio et al. (2013)	Cortisol	Saliva/SCL (high sensitivity electrochemiluminescence)		The more severe the periodontitis, the higher the cortisol levels (OR for periodontitis by cortisol levels 4.14 (95% CI 1.43–12.01)	
Rodriguez Franco et al. (2020)	IL1b, IL6, MMP- (matrix metalloproteinase)8	Saliva/ELISA	Depressive symptoms unrelated to proinflammatory immune response	Clinical attachment loss in IPD was associated with pro-inflammatory immune response (a composite of IL-1β, IL-6, MMP-8)	The activation of pro-inflammatory immune parameters in periodontal damage was independent of depression in their predictive (hypothetical) models.
Rosania et al. (2009)	Cortisol	Saliva/RIA (radioimmunoassay)	Cortisol levels alone not associated with depression score.	Positive correlation between Cortisol and higher degrees of periodontal disease measures (probing depth, tooth loss, CAL). Stress and cortisol levels were predictors of attachment loss.	Cortisol & depression in a regression model involving stress were significant predictors of clinical attachment loss
Solis et al. (2016)	IL6, IL-1β, INF-γ	GCF, whole blood, stimulated WBC/ELISA	Blood IL-6 and IL-1β and GCF IL-1β were modestly lower in MDD patients compared to controls. WBC upon LPS stimulation showed no differences in cytokine levels between MD and no MD group.		Cytokine differences in depression were independent of periodontal disease, no mediation analysis was available for IPD-MD associations

***Case-control studies (Human)***					
Bawankar et al. (2018)	Cortisol, IL-1β	Serum, saliva/ELISA		Salivary cortisol significantly higher in IPD group compared to healthy patient controls (417 ​± ​100 vs 20 ​± ​4 ​pg/ml). Higher serum (19 ​± ​6 vs. 11 ​± ​3 and salivary 251 ​± ​81 vs 160 ​± ​62 ​pg/ml. IL-β levels in IPD patients compared to healthy patient group. Serum cortisol levels not different between IPD and no IPD.	
Cohen-Cole et al. (1983)	Cortisol	Serum, urine/not reported		No significant differences between IPD patients and controls on measures of growth hormone, prolactin, or spot urine catecho- lamines. IPD patients had depressed lymphocyte function, polymorphonuclear leukocyte phagocytosis and chemotaxis.	Elevated urine and serum cortisol and depression score in IPD patients compared to healthy controls, but no analysis of the three factors together.
Johannsen et al. (2006)	Cortisol, IL-1β, IL-6, MMP (matrix metalloproteinase)-8, MMP-9	GCF, saliva/ELISA, RIA (radioimmunoassay)	MD patients had higher GCF cortisol (3.5 ​± ​3.3) vs 0.3 ​± ​0.3 ​nmol/l) and IL-6 (2.03 ​± ​1.6 vs 0.79 ​± ​1.8 pg/site) compared to controls (p ​< ​0.05). MD patients had lower MMP-9 (19.4 ​± ​12.1 vs 30.6 ​± ​18.5 ng/site) but GCF IL-1β and salivary cortisol not different between groups.		
Johannsen et al. (2007)	Cortisol, IL-1β, IL-6, MMP (matrix metalloproteinase)-8, MMP-9	GCF, saliva/ELISA, RIA (radioimmunoassay)	Higher GCF IL-6 level in MD vs controls (3.8 ​± ​1.6 pg/site vs 0.79 ​± ​1.83 pg/site, p ​< ​0.003), but no difference for IL-1β, MMP8, MMP-9 or salivary cortisol. Patients with MD had lower GCF cortisol than controls.		
Karimi et al. (2017)	IgA	Saliva		Salivary IgA level was lower in patients with periodontal disease (207.9 ​+ ​57.2) vs controls (312.66 ​+ ​107.3 units) (P ​= ​0.001).	
Leira et al. (2019)	CGRP (Calcitonin gene-related peptide), IL-6, IL-10	Serum/ELISA		IPD (with migraine) was associated with higher serum CGRP levels (19.7 ​± ​6.5 vs 15.3 ​± ​6.2 ​pg/mL, P ​< ​0.0001) and IL-6 (15.1 ​± ​9.2 vs 9.6 ​± ​6.3 ​pg/mL, P ​< ​0.0001), independent of MD. IL-10 did not show a difference.	IPD (with migraine) was associated with higher serum CGRP levels (19.7 ​± ​6.5 versus 15.3 ​± ​6.2 ​pg/mL, P ​< ​0.0001) and IL-6 (15.1 ​± ​9.2 versus 9.6 ​± ​6.3 ​pg/mL, P ​< ​0.0001), independent of depression.
Moss et al. (1996)	Antibodies (igG) against Bf, Aa, Pg	Blood/Antibody assay		IgG against *Porphyromonas gingivalis* and *Actinobacillus actinomycetemcomitans*) were strongly associated with Periodontitis (OR 4.54 (95% CI 2–10) and 5.3 (95%CI 2–12).	IgG against *Bacterioides forsythus* was associated with periodontal disease only among individuals with higher depression scores (OR 6.75 (95% CI 1.3–36.5). Periodontal pathogens related to depression. *P. gingivalis, Actinobacillus actinomycetemcomitans*. IgG against Bacteroides forsythus specifically related to periodontitis patients with depression.

***Prospective cohort studies (Human)***					
Cakmak et al. (2019)	Cortisol, DHEA	GCF/ELISA		Higher cortisol (pg/ml) levels in localized (338.2 ​± ​309) and generalized (388.0 ​± ​368) chronic periodontitis compared to patient controls (81.4 ​± ​27) p ​< ​0.001. No difference across groups at 6-month follow up, nor was DHEA levels different between the groups at baseline and follow up.	
Kurer et al. (1995)	Cortisol	saliva/RIA	No associations between cortisol level and depression score	No associations between cortisol level and plaque or gingivitis.	
Nascimento et al. (2019)	CRP	serum		No significant differences between CRP levels of periodontitis patients and healthy controls, as well as between CRP levels of patients with depression versus non-depressed subjects.	Association between depressive symptoms and periodontitis was not mediated by systemic inflammation.
Petit et al. (2020)	Cortisol, Chromogranin-A	serum, plasma/immunoassay	Cortisol not associated with depression score on DASS42	Stable cortisol and chromogranin-A levels at baseline and 6 months of non-surgical periodontal treatment despite improvement in multiple measures of periodontal outcomes.	
Petit et al. (2021)	Cortisol, Chromogranin-A	plasma/immunoassay	No correlation between plasma cortisol or chromogranin-A with psychological status	No correlation between plasmatic cortisol or chromogranin-A with SRP outcomes	
Zhang et al. (2021)	Cortisol, Interleukin B	saliva/ELISA		No significant difference in cortisol or IL-1β levels between periodontitis patients and healthy controls.	IPD-patients (smokers) with depression had increased levels of both cortisol and interleukin B compared to IPD-only patients (non-smokers) and healthy controls.

Notes: IPD: inflammatory periodontal disease, MD: major depression, BDI: Beck Depression Inventory, HDRS: Hamilton Depression Rating Scale, BDNF: brain derived neurotrophic factor, CRP: C-reactive Protein, DHEA: dehydroepiandrosterone, iNOS: inducible NO synthase, GR receptor: glucocorticoid receptor, AOPP: advanced oxidation protein products, NOx: nitric oxide metabolites, LPS: lipopolysaccharide, LOOH: lipid peroxides, MMP: matrix metalloproteinase, –SH: sulfhydryl groups, TRAP: total radical trapping antioxidant parameter, PON1: paraoxonase, WBC: white blood cells, CAL: clinical attachment level, PPD: probing pocket depth, GCF: gingival crevicular fluid.

### 3.4 Biomarkers by IPD and MD

Two studies that used animal models for periodontal disease found eleveated inflammatory signaling and reduced neurotrophic support in the brain compared to that of control animals as indicated by higher TNF-α, IL-1β, IL-6 and TLR-4, increased number of activated glial cells as well as reduced BDNF (brain-derived neurotrophic factor) levels (Martínez et al., 2021; Wang et al., 2019). Similar findings were reported for depression-like behavior in animals (Martínez et al., 2021; Wang et al., 2019). Glucocorticoid receptor expression was reported to be decreased in the hippocampus of depression model rats relative to controls (Breivik et al., 2006, 2015).

Twenty of the included studies on human subjects reported on biomarkers related to periodontal disease, fiftteen of which found an association between the measured biomarkers and periodontitis. Most studies reported cortisol levels measured in saliva, GCF, serum or urine to be positively correlated with periodontal disease (Bawankar et al., 2018; Cakmak et al., 2014, 2016; Cohen-Cole et al., 1983; Fenol et al., 2017; Rahate et al., 2021; Refulio et al., 2013; Rosania et al., 2009). However, five studies found no associations between salivary cortisol and periodontal disease (Cakmak et al., 2019; da Silva et al., 2015; Kurer et al., 1995; Petit et al., 2020; Zhang et al., 2021). While Zhang et al. (2021) observed no significant difference in salivary IL-1β, Bawankar et al. (2018) found higher serum and salivary IL-1β levels in periodontal patients.

Studies reporting on the GCF and salivary levels of cortisol, MMP (matrix metalloproteinase)-8, MMP-9 showed mixed associations with depression (Johannsen et al., 2006, 2007). Katuri et al. (2016) reported on increased levels of serum cortisol in the depressed group. Solis et al. (2016) reported modestly lower levels of IL-6 and IL-1β in blood, and IL-1β in GCF in patients with depression. A significant association between clinical depression and increased levels of root canal LPS, plasma AOPP (advanced oxidation protein products), NOx (nitric oxide metabolites), LOOH (lipid peroxides), and TRAP (total radical trapping antioxidant parameter) was found (Gomes et al., 2018). Depression was not associated with analyzed biomarkers, including cortisol, in five studies (Kurer et al., 1995; Petit et al., 2020, 2021; Rodriguez Franco et al., 2020; Rosania et al., 2009).

### 3.5 Biomarkers in the relationship between IPD and MD

In animal studies, several of the analyzed biomarkers were related to co-occurrence of periodontitis and depression. Wang et al. (2019) found reduced levels of mature BDNF mediated by increased levels of astrocytic p75NTR in a mouse periodontitis model resulting in depression like behavior (Wang et al., 2019). Breivik et al. (2006) found significantly higher serum levels of cortisol and TGF-1β, and decreased TNF-α levels in a rat depression model with ligature-induced periodontitis compared to IPD only rats (Breivik et al., 2006). Rats presenting both with depression and periodontitis also had significantly higher levels of serum cortisol following LPS stimulation. Furthermore, the same group reported higher hippocampal GR expression in depressed rats with periodontitis, even though there was no association between levels of cortisol, IL-10 or TNF-α and depression (Breivik et al., 2015). Martínez et al. (2021) investigated the expression of a plethora of pro-inflammatory markers including TNF-α, NF-kB, IL-1β, iNOS, mPGES, TLR-4 and markers involved in neuroinflammation (APOA1, corticosterone and GR expression) in the brain, and found several to be dysregulated in depressed/stressed IPD rats compared to controls.

Systemic and urinary cortisol were associated with elevated depression scores in patients with acute necrotizing ulcerative gingivitis (ANUG) (Cohen-Cole et al., 1983). One study concluded that IgG against *Bacterioides forsythus (Bf)* was associated with periodontal disease only in subjects with higher depression scores (Moss et al., 1996). Increased levels of root canal LPS were recorded in patients with chronic apical periodontitis and depression (Gomes et al., 2018). One study reported stress (including depression) and cortisol levels as predictors of attachment loss (Rosania et al., 2009). Patients with both depression and IPD presented with increased levels of cortisol and IL-1β in another study (Zhang et al., 2021). In the study (da Silva et al., 2015), there was a strong correlation between MD and biomarker (diurnal decline in salivary cortisol) in IPD group (r ​= ​−0.64; p ​< ​0.01), but not in control group (r ​= ​0.07, NS). Also Rahate et al. (2021) reported on increased levels of salivary cortisol in depressed IPD patients compared to IPD-only patients.

Other studies did not find common biomarkers to explain the link between depression and IPD. Solis et al. (2016) found that the lower levels of cortisol seen in patients with MD were independent of periodontal status. One study concluded that the association between periodontal disease and depressive symptoms is not mediated by systemic inflammation (Nascimento et al., 2019). The activation of proinflammatory immune parameters (IL-1β, IL-6, MMP-8) in periodontal damage was found to be independent of depression in a predictive (hypothetical) model among humans (Rodriguez Franco et al., 2020).

## 4 Discussion

We examined available literature for the association between depression and periodontitis putatively mediated or related to shared biomarkers. Out of 28 included studies, fourteen human and four animal studies found a positive association between IPD and MD while the rest reported no such associations. A previous systematic review on the relationship between periodontitis and depression selected 15 relevant studies out of which only six concluded on the positive association between these conditions (Araújo et al., 2016), pointing at some progress in this research field. Nevertheless, in our review, we focused on the importance of biomarkers linking the two conditions and not just on their association. Taken separately, most studies found links between periodontal inflammation and cortisol or other biomarker levels, as well as depression and inflammatory markers; yet, in the IPD-MD connection, only ten studies reported on the association of the two conditions via an inflammatory element. Out of these, four studies were performed on animals.

In the current review, animal studies (Breivik et al., 2006, 2015; Martínez et al., 2021; Wang et al., 2019) support the link between depression and periodontal disease via inflammatory biomarkers. This may be due to the ability to subject all study participants to standardized conditions and disease exposures. Nevertheless, there are differences in the presented results. In 2006, Breivik et al. (2006) induced depression by olfactory bulbectomy (OB), while in 2015 the same group used the maternal deprivation paradigm for the same purpose. All rats with depression developed a more severe periodontal disease. Yet, in the OB model, rats presented with a decreased glucocorticoid receptor (GR) expression in the hippocampus, while maternally-deprived animals showed increased hippocampal GR expression. This may be indicative of the different degrees of stress and depression that can result in dissimilar biomarker levels that we also see in human subjects. It is considered that an early onset of stressful events, such as in the maternal deprivation model, is thought to persistently change the reactivity to stressors, including immune responses to pathogens (McEwen et al., 2012; Weaver, 2009). This epigenetic modulation of adult human subjects from mixed backgrounds, countries and populations that are included in studies on depression and periodontal disease is difficult to interpret and be adjusted for, contributing to the conflicting results. Based on the available data, it seems plausible that disrupted hypothalamic–pituitary–adrenal axis (HPA axis) and glucocorticoid resistance (i.e., reduced function of glucocorticoid receptor) may directly compromise immune function contributing to the neuroimmune-endocrine pathogenesis of depression (Perrin et al., 2019), as reflected in altered neuroimmune and neurotrophic factors in multiple studies in this review.

The possibility of inflammation and depression being related was out of the question a few decades ago (Beurel et al., 2020; Dantzer, 2012). The emergence of psychoneuroimmunology (Ader and Cohen, 1975) and the observed dysregulation in the immune system seen in depressed patients indicated the need for a different approach to the condition (Stein et al., 1985). Elevated levels of IL-6, IL-10, TNF-α or CRP have been associated with depression in different studies (Chamberlain et al., 2019; Irwin and Miller, 2007; Köhler et al., 2017).

Nevertheless, the questions raised are still a matter of debate: is the inflammation primary or does depression promote inflammation? One prospective study on young individuals did indicate that depression episodes increased CRP levels and as such may be considered an inflammatory promoter (Copeland et al., 2012). More evidence is suggesting the role of inflammation in subsequent development of depression (Bondy et al., 2021). Still, a range of other comorbid factors are accountable for an increase in systemic inflammation in patients with depression. Those factors include obesity, smoking, immobility, dysbiotic gut, dental cares or sleep disturbance (Berk et al., 2013). Periodontitis stands out as one such potential factors that has received little attention.

Bacteria are the major culprits in the development of periodontits, but the tissue response to this aggression results in the secretion and interaction of a plethora of inflammatory mediators contributing to the pathogenesis of periodontal disease (Freire et al., 2021; Suárez et al., 2020). Knowing more on how innate and adaptive immune responses function indicates that the condition is not just a localized pathological event. These biomarkers have been related to the systemic impact of periodontitis and its possible association with other conditions (Esteves Lima et al., 2013). At the same time, psychological stress and ineffective coping can also influence the onset and progression of many chronic diseases, including periodontitis through immune suppression (Ng and Keung Leung, 2006; Petit et al., 2020; Zhang et al., 2021). We must not forget that health-related behaviors such as oral hygiene, diet and smoking may be modified by stress response and depression to favor oral inflammation (Aleksejuniené et al., 2002; Genco et al., 1998). While this two-way association has been shown for decades, the bi-directional relationship between depression and periodontal disease and a possible causality has been difficult to establish (Genco et al., 1999; Monteiro da Silva et al., 1996; Moss et al., 1996; Winning and Linden, 2017). The search for diagnostic disease biomarkers has led to the analysis of different cytokines or hormones. One of the most analyzed biomarkers in the context of depression or periodontal disease is cortisol. The results on a positive association between cortisol and periodontitis or depression are conflicting. It appears that cortisol dysregulation, specifically in response to stress, is reliably associated with severe forms of depression. However, chronic and less severe types of depression do not share the same robust association (Angst et al., 2007).

As such, this may explain why the study by (Rosania et al., 2009) did not find an association between depression scores and cortisol levels in patients who self-evaluated the disorder with questions referring mostly to last week symptoms. Stress in non-depressed individuals is known to increase inflammatory markers. Recent evidence suggests that depressed individuals not only have increased baseline inflammation but also show an exaggerated inflammatory response to stress and a diminished responsivity to cortisol. The study by Rosania et al. did use a regression model in which stress was the predictor; in that model, cortisol and stress were shown to be predictors of CAL. In the same context, Petit et al. (Petit et al., 2020, 2021) have concluded that stress associated positively with poor outcomes of periodontal treatment. However, cortisol levels could not be associated with depression scores, nor with periodontal status. Nevertheless, the effects of oral hygiene or periodontal treatments may not be reflective in biomarker levels due to the studied populations; recruited subjects in these studies are adherent to a periodontal maintenance program (Petit et al., 2020; Rosania et al., 2009). Similarly, Kurer et al. (1995) only included patients with good oral health and could not associate cortisol levels to depression or periodontal inflammation. Smoking is a major contributor to both immune and comorbid psychopathologies, requiring a close consideration in all biological assessments of IPD-MD links (Moeintaghavi et al., 2017), which was also reflected in some of the included studies that performed separate analyses for smokers and non-smokers (Bawankar et al., 2018; Rahate et al., 2021; Zhang et al., 2021).

Solis et al. (2016) examined IL-6, IL-1β and INF-γ in GCF as well as systemically and could not prove the hypothesis that immunological activation during depressive episodes contributes to increased levels of cytokines. A certain immunosuppression in MD patients was seen, however. One explanation may be that the sample sizes were quite small, and that types of depression may not be independently distinguished.

Several other methodological limitations of these evaluated studies should be named. First, the majority of studies are observational. It is known that these designs come with the great weakness of not being able to protect against confounding. While most of them used statistical techniques to adjust for possible confounders, there may be residual unknown or unrecognized factors that remain in a heterogeneous adult study population (Smith and Ebrahim, 2003). This is a problem for studies investigating exposures such as depression and periodontitis which can be affected by factors such as health service utilization or socioeconomic status. Cohen-Cole et al. (1983) did find an association between ANUG, depression and elevated cortisol levels; however, the studied population was small, men were underrepresented, and was predominantly of low income. Furthermore, the study design was retrospective. While a statistical adjustment for low income is stated, could other factors related to stressful life events influence the disease progression in the analyzed study population? As such, a causality cannot be concluded, even though an association between depression, acute periodontal inflammation and cortisol levels is noted.

Another critical aspect for the analyzed studies is the big age-span of included subjects. Even though the quality of studies increases if a statistical adjustment is included, other possible unknown age-related confounding factors, particularly multimorbidities associated with immune inflammatory alterations, may influence the outcome of an investigation.

The conflicting results between the investigations may also be explained by the differences between the different levels of periodontal disease severity and the high variation of used clinical parameters. It is also the big variability in studied biomarkers along with testing methodology and limited number of studies for shared biomarkers using the same biological sample that hampered the meta-analysis of existing literature.

In conclusion, there is a substantial data supporting associations between periodontal disease and depression and inflammatory contribution to each disorder. However, limited evidence is available on the role of biomarkers in the possible causal pathway between depression and periodontal disease. This is generated by the high heterogeneity among the type of study, the included populations, the assay methods, and the evaluated biomarkers. Future randomized control and prospective studies with standardized clinical and biological assessments are required to establish causality between periodontal inflammation and depression and further analyze the role of biomarkers in linking these diseases.

## Funding

This study was funded through internal resources at the affiliated institutions.

## Declaration of competing interest

None.

## References

[bib78] Ader R., Cohen N. (1975). Behaviorally conditioned immunosuppression. Psychosom. Med..

[bib1] Aldosari M., Helmi M., Kennedy E.N., Badamia R., Odani S., Agaku I., Vardavas C. (2020). Depression, periodontitis, caries and missing teeth in the USA, NHANES 2009-2014. Fam. Med. Commun. Health.

[bib2] Aleksejuniené J., Holst D., Eriksen H.M., Gjermo P. (2002). Psychosocial stress, lifestyle and periodontal health. J. Clin. Periodontol..

[bib3] Angst J., Gamma A., Benazzi F., Ajdacic V., Rössler W. (2007). Melancholia and atypical depression in the Zurich study: epidemiology, clinical characteristics, course, comorbidity and personality. Acta Psychiatr. Scand..

[bib4] Araújo M.M., Martins C.C., Costa L.C., Cota L.O., Faria R.L., Cunha F.A., Costa F.O. (2016). Association between depression and periodontitis: a systematic review and meta-analysis. J. Clin. Periodontol..

[bib5] Bawankar P.V., Kolte A.P., Kolte R.A. (2018). Evaluation of stress, serum and salivary cortisol, and interleukin-1beta levels in smokers and non-smokers with chronic periodontitis. J. Periodontol..

[bib6] Berk M., Williams L.J., Jacka F.N., O'Neil A., Pasco J.A., Moylan S., Allen N.B., Stuart A.L., Hayley A.C., Byrne M.L., Maes M. (2013). So depression is an inflammatory disease, but where does the inflammation come from?. BMC Med.

[bib7] Beurel E., Toups M., Nemeroff C.B. (2020). The bidirectional relationship of depression and inflammation: double trouble. Neuron.

[bib8] Bondy E., Norton S.A., Voss M., Marks R.B., Boudreaux M.J., Treadway M.T., Oltmanns T.F., Bogdan R. (2021). Inflammation is associated with future depressive symptoms among older adults. Brain Behav. Immun. Health.

[bib9] Breivik T., Gundersen Y., Murison R., Turner J.D., Muller C.P., Gjermo P., Opstad K. (2015). Maternal deprivation of lewis rat pups increases the severity of experi-mental periodontitis in adulthood. Open Dent. J..

[bib10] Breivik T., Gundersen Y., Myhrer T., Fonnum F., Osmundsen H., Murison R., Gjermo P., von Horsten S., Opstad P.K. (2006). Enhanced susceptibility to periodontitis in an animal model of depression: reversed by chronic treatment with the anti-depressant tianeptine. J. Clin. Periodontol..

[bib11] Cakmak O., Alkan B.A., Ozsoy S., Sen A., Abdulrezzak U. (2014). Association of gingival crevicular fluid cortisol/dehydroepiandrosterone levels with periodontal status. J. Periodontol..

[bib12] Cakmak O., Alkan B.A., Saatci E., Tasdemir Z. (2019). The effect of nonsurgical periodontal treatment on gingival crevicular fluid stress hormone levels: a prospective study. Oral Dis.

[bib13] Cakmak O., Tasdemir Z., Aral C.A., Dundar S., Koca H.B. (2016). Gingival crevicular fluid and saliva stress hormone levels in patients with chronic and aggressive periodontitis. J. Clin. Periodontol..

[bib14] Chamberlain S.R., Cavanagh J., de Boer P., Mondelli V., Jones D.N.C., Drevets W.C., Cowen P.J., Harrison N.A., Pointon L., Pariante C.M., Bullmore E.T. (2019). Treatment-resistant depression and peripheral C-reactive protein. Br. J. Psychiatry.

[bib15] Choi J., Price J., Ryder S., Siskind D., Solmi M., Kisely S. (2021). Prevalence of dental disorders among people with mental illness: an umbrella review. Aust. N. Z. J. Psychiatr..

[bib16] Cohen-Cole S.A., Cogen R.B., Stevens A.W., Kirk K., Gaitan E., Bird J., Cooksey R., Freeman A. (1983). Psychiatric, psychosocial, and endocrine correlates of acute necrotizing ulcerative gingivitis (trench mouth): a preliminary report. Psychiatr. Med..

[bib17] Copeland W.E., Shanahan L., Worthman C., Angold A., Costello E.J. (2012). Cumulative depression episodes predict later C-reactive protein levels: a prospective analysis. Biol. Psychiatr..

[bib18] da Silva P.D., Barbosa T.D., Amato J.N., Montes A.B.M., Gaviao M.B.D. (2015). Gingivitis, psychological factors and quality of life in children. Oral Health Prev. Dent..

[bib19] Dantzer R. (2012). Depression and inflammation: an intricate relationship. Biol. Psychiatr..

[bib20] Esteves Lima R.P., Miranda Cota L.O., Costa F.O. (2013). Association between periodontitis and gestational diabetes mellitus: a case-control study. J. Periodontol..

[bib21] Fenol A., Jebi S., Krishnan S., Perayil J., Vyloppillil R., Bhaskar A., Menon S., Mohandas A. (2017). Association of stress, salivary cortisol level, and periodontitis among the inmates of a central prison in Kerala. Dent. Res. J..

[bib22] Freire M., Nelson K.E., Edlund A. (2021). The oral host-microbial interactome: an ecological chronometer of health?. Trends Microbiol.

[bib23] Genco R.J., Ho A.W., Grossi S.G., Dunford R.G., Tedesco L.A. (1999). Relationship of stress, distress and inadequate coping behaviors to periodontal disease. J. Periodontol..

[bib24] Genco R.J., Ho A.W., Kopman J., Grossi S.G., Dunford R.G., Tedesco L.A. (1998). Models to evaluate the role of stress in periodontal disease. Ann. Periodontol..

[bib25] Gomes C., Martinho F.C., Barbosa D.S., Antunes L.S., Povoa H.C.C., Baltus T.H.L., Morelli N.R., Vargas H.O., Nunes S.O.V., Anderson G., Maes M. (2018). Increased root canal endotoxin levels are associated with chronic apical periodontitis, increased oxidative and nitrosative stress, major depression, severity of depression, and a lowered quality of life. Mol. Neurobiol..

[bib26] Goyal S., Jajoo S., Nagappa G., Rao G. (2011). Estimation of relationship between psychosocial stress and periodontal status using serum cortisol level: a clinico-biochemical study. Indian J. Dent. Res..

[bib27] Hashioka S., Inoue K., Miyaoka T., Hayashida M., Wake R., Oh-Nishi A., Inagaki M. (2019). The possible causal link of periodontitis to neuropsychiatric disorders: more than psychosocial mechanisms. Int. J. Mol. Sci..

[bib28] Hilgert J.B., Hugo F.N., Bandeira D.R., Bozzetti M.C. (2006). Stress, cortisol, and periodontitis in a population aged 50 years and over. J. Dent. Res..

[bib29] Hooijmans C.R., Rovers M.M., de Vries R.B., Leenaars M., Ritskes-Hoitinga M., Langendam M.W. (2014). SYRCLE's risk of bias tool for animal studies. BMC Med. Res. Methodol..

[bib30] Hsu C.C., Hsu Y.C., Chen H.J., Lin C.C., Chang K.H., Lee C.Y., Chong L.W., Kao C.H. (2015). Association of periodontitis and subsequent depression: a nationwide population-based study. Medicine (Baltim.).

[bib31] Irwin M.R., Miller A.H. (2007). Depressive disorders and immunity: 20 years of progress and discovery. Brain Behav. Immun..

[bib32] Ishisaka A., Ansai T., Soh I., Inenaga K., Awano S., Yoshida A., Hamasaki T., Sonoki K., Takata Y., Nishihara T., Takehara T. (2008). Association of cortisol and dehydroepiandrosterone sulphate levels in serum with periodontal status in older Japanese adults. J. Clin. Periodontol..

[bib33] Johannsen A., Rydmark I., Soder B., Asberg M. (2007). Gingival inflammation, increased periodontal pocket depth and elevated interleukin-6 in gingival crevicular fluid of depressed women on long-term sick leave. J. Periodontal. Res..

[bib34] Johannsen A., Rylander G., Soder B., Asberg M. (2006). Dental plaque, gingival inflammation, and elevated levels of interleukin-6 and cortisol in gingival crevicular fluid from women with stress-related depression and exhaustion. J. Periodontol..

[bib35] Kurer J.R., Watts T.L., Weinman J., Gower D.B. (1995). Psychological mood of regular dental attenders in relation to oral hygiene behaviour and gingival health. J. Clin. Periodontol..

[bib79] Karimi M., Elyahoo S., Golchin L., Kermani T. (2017). Relationship between stress, anxiety, depression and salivary IgA with periodontal disease. Biosci. Biotechnol. Res. Commun..

[bib77] Katuri K.K., Dasari A.B., Kurapati S., Vinnakota N.R., Bollepalli A.C., Dhulipalla R. (2016). Association of yoga practice and serum cortisol levels in chronic periodontitis patients with stress-related anxiety and depression. J. Int. Soc. Prev. Community Dent..

[bib36] Köhler C.A., Freitas T.H., Maes M., de Andrade N.Q., Liu C.S., Fernandes B.S., Stubbs B., Solmi M., Veronese N., Herrmann N., Raison C.L., Miller B.J., Lanctôt K.L., Carvalho A.F. (2017). Peripheral cytokine and chemokine alterations in depression: a meta-analysis of 82 studies. Acta Psychiatr. Scand..

[bib81] Leira Y., Ameijeira P., Domínguez C., López-Arias E., Ávila-Gómez P., Pérez-Mato M., Sobrino T., Campos F., D’Aiuto F., Leira R., Blanco J. (2019). Periodontal inflammation is related to increased serum calcitonin gene-related peptide levels in patients with chronic migraine. J. Periodontol..

[bib37] Mac Giollabhui N., Ng T.H., Ellman L.M., Alloy L.B. (2021). The longitudinal associations of inflammatory biomarkers and depression revisited: systematic review, meta-analysis, and meta-regression. Mol. Psychiatr..

[bib38] Maes M., Galecki P., Chang Y.S., Berk M. (2011). A review on the oxidative and nitrosative stress (O&NS) pathways in major depression and their possible contribution to the (neuro)degenerative processes in that illness. Prog. Neuro-Psychopharmacol. Biol. Psychiatr..

[bib39] Maier S.F., Watkins L.R. (1998). Cytokines for psychologists: implications of bidirectional immune-to-brain communication for understanding behavior, mood, and cognition. Psychol. Rev..

[bib40] Mariani N., Cattane N., Pariante C., Cattaneo A. (2021). Gene expression studies in Depression development and treatment: an overview of the underlying molecular mechanisms and biological processes to identify biomarkers. Transl. Psychiatry.

[bib41] Martínez M., Martín-Hernández D., Virto L., MacDowell K.S., Montero E., González-Bris Á., Marín M.J., Ambrosio N., Herrera D., Leza J.C., Sanz M., García-Bueno B., Figuero E. (2021). Periodontal diseases and depression: a pre-clinical in vivo study. J. Clin. Periodontol..

[bib42] McEwen B.S., Eiland L., Hunter R.G., Miller M.M. (2012). Stress and anxiety: structural plasticity and epigenetic regulation as a consequence of stress. Neuropharmacology.

[bib43] Moeintaghavi A., Arab H.R., Rahim Rezaee S.A., Naderi H., Shiezadeh F., Sadeghi S., Anvari N. (2017). The effects of smoking on expression of IL-12 and IL-1β in gingival tissues of patients with chronic periodontitis. Open Dent. J..

[bib44] Moher D., Shamseer L., Clarke M., Ghersi D., Liberati A., Petticrew M., Shekelle P., Stewart L.A. (2015). Preferred reporting items for systematic review and meta-analysis protocols (PRISMA-P) 2015 statement. Syst. Rev..

[bib45] Monteiro da Silva A.M., Oakley D.A., Newman H.N., Nohl F.S., Lloyd H.M. (1996). Psychosocial factors and adult onset rapidly progressive periodontitis. J. Clin. Periodontol..

[bib46] Moss M.E., Beck J.D., Kaplan B.H., Offenbacher S., Weintraub J.A., Koch G.G., Genco R.J., Machtei E.E., Tedesco L.A. (1996). Exploratory case-control analysis of psychosocial factors and adult periodontitis. J. Periodontol..

[bib47] Nascimento G.G., Gastal M.T., Leite F.R.M., Quevedo L.A., Peres K.G., Peres M.A., Horta B.L., Barros F.C., Demarco F.F. (2019). Is there an association between depression and periodontitis? A birth cohort study. J. Clin. Periodontol..

[bib48] National Institutes of Health (2001). Biomarkers and surrogate endpoints: preferred definitions and conceptual framework. Clin. Pharmacol. Ther..

[bib49] Ng S.K., Keung Leung W. (2006). A community study on the relationship between stress, coping, affective dispositions and periodontal attachment loss. Community Dent. Oral Epidemiol..

[bib50] Osimo E.F., Baxter L.J., Lewis G., Jones P.B., Khandaker G.M. (2019). Prevalence of low-grade inflammation in depression: a systematic review and meta-analysis of CRP levels. Psychol. Med..

[bib51] Ouzzani M., Hammady H., Fedorowicz Z., Elmagarmid A. (2016). Rayyan-a web and mobile app for systematic reviews. Syst. Rev..

[bib52] Pan W., Wang Q., Chen Q. (2019). The cytokine network involved in the host immune response to periodontitis. Int. J. Oral Sci..

[bib53] Perrin A.J., Horowitz M.A., Roelofs J., Zunszain P.A., Pariante C.M. (2019). Glucocorticoid resistance: is it a requisite for increased cytokine production in depression? A systematic review and meta-analysis. Front. Psychiatr..

[bib54] Peruzzo D.C., Benatti B.B., Ambrosano G.M., Nogueira-Filho G.R., Sallum E.A., Casati M.Z., Nociti F.H. (2007). A systematic review of stress and psychological factors as possible risk factors for periodontal disease. J. Periodontol..

[bib55] Petit C., Anadon-Rosinach V., Rettig L., Schmidt-Mutter C., Tuzin N., Davideau J.L., Huck O. (2020). Influence of psychological stress on non-surgical periodontal treatment outcomes in patients with severe chronic periodontitis. J. Periodontol..

[bib56] Petit C., Anadon-Rosinach V., Rettig L., Schmidt-Mutter C., Tuzin N., Davideau J.L., Huck O. (2021). Influence of psychological stress on non-surgical periodontal treatment outcomes in patients with severe chronic periodontitis. J. Periodontol..

[bib57] Quan N., Banks W.A. (2007). Brain-immune communication pathways. Brain Behav. Immun..

[bib58] Rahate P.S., Kolte R.A., Kolte A.P., Lathiya V.N., Gupta M., Chari S. (2021). Evaluation of stress, serum, and salivary ghrelin and cortisol levels in smokers and non-smokers with Stage III periodontitis-A cross-sectional study. J. Periodontol..

[bib59] Raison C.L., Capuron L., Miller A.H. (2006). Cytokines sing the blues: inflammation and the pathogenesis of depression. Trends Immunol..

[bib60] Raison C.L., Miller A.H. (2011). Is depression an inflammatory disorder?. Curr. Psychiatr. Rep..

[bib61] Raison C.L., Miller A.H. (2013). Role of inflammation in depression: implications for phenomenology, pathophysiology and treatment. Modern Trends pharmacopsychiatr..

[bib62] Refulio Z., Rocafuerte M., de la Rosa M., Mendoza G., Chambrone L. (2013). Association among stress, salivary cortisol levels, and chronic periodontitis. J. Periodontal Implant Sci..

[bib63] Rodriguez Franco N.I., Moral de la Rubia J., Alcazar Pizana A.G. (2020). Predictive model of clinical attachment loss and oral health-related quality of life through depressive symptomatology, oral hygiene habits, and proinflammatory biomarkers: a pilot study. Dent. J..

[bib64] Rosania A.E., Low K.G., McCormick C.M., Rosania D.A. (2009). Stress, depression, cortisol, and periodontal disease. J. Periodontol..

[bib65] Smith G.D., Ebrahim S. (2003). Mendelian randomization': can genetic epidemiology contribute to understanding environmental determinants of disease?. Int. J. Epidemiol..

[bib66] Solis A.C.O., Marques A.H., Dominguez W.V., Prado E.B.A., Pannuti C.M., Lotufo R.F.M., Lotufo-Neto F. (2016). Evaluation of periodontitis in hospital outpatients with major depressive disorder. A focus on gingival and circulating cytokines. Brain Behav. Immun..

[bib80] Solis A.C., Marques A.H., Pannuti C.M., Lotufo R.F., Lotufo-Neto F. (2014). Evaluation of periodontitis in hospital outpatients with major depressive disorder. J. Periodontal. Res..

[bib67] Stein M., Keller S.E., Schleifer S.J. (1985). Stress and immunomodulation: the role of depression and neuroendocrine function. J. Immunol..

[bib68] Suárez L.J., Garzón H., Arboleda S., Rodríguez A. (2020). Oral dysbiosis and autoimmunity: from local periodontal responses to an imbalanced systemic immunity. A review. Front. Immunol..

[bib69] Wang Y.X., Kang X.N., Cao Y., Zheng D.X., Lu Y.M., Pang C.F., Wang Z., Cheng B., Peng Y. (2019). Porphyromonas gingivalis induces depression via downregulating p75NTR-mediated BDNF maturation in astrocytes. Brain Behav. Immun..

[bib70] Warren K.R., Postolache T.T., Groer M.E., Pinjari O., Kelly D.L., Reynolds M.A. (2014). Role of chronic stress and depression in periodontal diseases. Periodontol. 2000.

[bib71] Watkins L.R., Maier S.F., Goehler L.E. (1995). Cytokine-to-brain communication: a review & analysis of alternative mechanisms. Life Sci..

[bib72] Weaver I.C. (2009). Shaping adult phenotypes through early life environments. Birth Defects Res. C Embryo Today.

[bib73] Winning L., Linden G.J. (2017). Periodontitis and systemic disease: association or causality?. Curr. Oral Health Rep..

[bib74] Zaric S., Shelburne C., Darveau R., Quinn D.J., Weldon S., Taggart C.C., Coulter W.A. (2010). Impaired immune tolerance to Porphyromonas gingivalis lipopolysaccharide promotes neutrophil migration and decreased apoptosis. Infect. Immun..

[bib75] Zhang H., Chen B., Pan C., Zhang A. (2021). To evaluate the serum cortisol, salivary cortisol, and serum interleukin-1 B level in patients of chronic periodontitis with smoking and stress and without smoking and stress. Medicine (Baltim.).

[bib76] Zheng D.X., Kang X.N., Wang Y.X., Huang Y.N., Pang C.F., Chen Y.X., Kuang Z.L., Peng Y. (2021). Periodontal disease and emotional disorders: a meta-analysis. J. Clin. Periodontol..

