# Supplementary material for: Biomarkers common for inflammatory periodontal disease and depression: A systematic review
Source: Brain Behav Immun Health. 2022 Mar 14;21:100450. doi: 10.1016/j.bbih.2022.100450 (PMC8938251; doi:10.1016/j.bbih.2022.100450)
Supplement: Multimedia component 1 [file mmc1.docx]

**Appendix 1.0 Search strategy**

*Database*: The following electronic databases shall be searched: MEDLINE, PsycINFO, Web of Science, Scopus, and Embase. We will also check bibliographies of the published meta-analyses for inclusion criteria. Grey literature shall not be considered.

Search terms: The search terms will be based on the MESH terms that cover variants of the key words and concepts: gingivitis, inflammatory periodontal diseases, periodontitis, gingival hemorrhage AND depression or depressive disorder. Only studies that used a biomarker to assess associations between depression and the above-mentioned oral pathologies will be included.

Copy of searches:

Before deduplication: 3010 references

After deduplication: 1361 references

**Embase, Ovid MEDLINE(R), APA PsycInfo**

Embase Classic+Embase 1947 to 2020 November 25, Ovid MEDLINE(R) ALL 1946 to November 25, 2020, APA PsycInfo 1806 to November Week 3 2020

Date of search: 2020-11-27
Retrieved references: Embase 1033, Medline 436, PsycInfo 31

| 1 | (gingivitis/ or gingivitis, necrotizing ulcerative/ or peri-implantitis/ or exp periapical periodontitis/ or periodontitis/ or aggressive periodontitis/ or chronic periodontitis/ or periapical periodontitis/ or periodontal abscess/ or periodontal pocket/ or Pericoronitis/ or Gingival Hemorrhage/) use medall | 41190 |
| --- | --- | --- |
| 2 | (gingiva disease/ or gingivitis/ or periodontitis/ or aggressive periodontitis/ or chronic periodontitis/ or periodontal disease/ or periimplantitis/ or gingiva disease/ or gingiva bleeding/) use emczd | 95211 |
| 3 | (gingivitis or periodontitis or periodontal disease* or periodontal inflammation* or peri-implantitis or Gingival disease* or Gingival inflammation* or pericoronitis or gum bleed* or dental bleed* or ((gum or dental or gingival or gingiva) adj3 (haemorrhage or hemorrhage or bleed*))).tw,kw,kf,hw,sh. | 184446 |
| 4 | 1 or 2 or 3 | 193524 |
| 5 | (Depression/ or exp Depressive Disorder/) use medall | 219903 |
| 6 | exp depression/ use emczd | 501840 |
| 7 | Depress*.tw,kf,kw. | 1470831 |
| 8 | (exp major depression/ or atypical depression/) use psyh | 134415 |
| 9 | 5 or 6 or 7 or 8 | 1679887 |
| 10 | 4 and 9   \| Embase Classic+Embase <1947 to 2020 November 25>  Ovid MEDLINE(R) ALL <1946 to November 25, 2020>  APA PsycInfo <1806 to November Week 3 2020> \| 1033  436  31 \| \| --- \| --- \| | 1500 |

Key: /=medical subject heading (MeSH) in Medline, /=EMTREE subject headings in EMBASE, / = The *PsycINFO* Thesaurus, tw= text words (words in title, abstract, key concepts in PsycInfo), kw = author supplied keywords in Embase, kf = author supplied keywords in Medline, sh=Medical Subject Headings (MeSH), ADJn = word distance of maximum n words.

**Cochrane Library**

Date of search: 2020-11-27
Retrieved references: 59 in Trials

| #1 | ([mh gingivitis] or [mh "gingivitis, necrotizing ulcerative"] or [mh "peri-implantitis"] or [mh "periapical periodontitis"] or [mh periodontitis] or [mh "aggressive periodontitis"] or [mh "chronic periodontitis"] or [mh "periapical periodontitis"] or [mh "periodontal abscess"] or [mh "periodontal pocket"] or [mh Pericoronitis] or [mh "Gingival Hemorrhage"]) | 4342 |
| --- | --- | --- |
| #2 | (gingivitis or periodontitis or periodontal disease* or periodontal inflammation* or peri-implantitis or Gingival disease* or Gingival inflammation* or pericoronitis or gum bleed* or dental bleed* or ((gum or dental or gingival or gingiva) NEAR/3 (haemorrhage or hemorrhage or bleed*))):ti,ab,kw | 10928 |
| #3 | #1 OR #2 | 11177 |
| #4 | ([mh Depression] or [mh "Depressive Disorder"]) | 20932 |
| #5 | depress*:ti,ab,kw | 85940 |
| #6 | #4 or #5 | 85996 |
| #7 | #3 and #6 | 59 |

Key: ti = title, ab = abstract, kw = keyword, [mh XX] = medical subject heading (MeSH).

**Scopus (Elsevier, 1788-2020)**
Date of search: 2020-11-27
Retrieved references: 1014

( TITLE-ABS-KEY ( ( gingivitis  OR  periodontitis  OR  "periodontal disease*"  OR  "periodontal inflammation*"  OR  peri-implantitis  OR  "Gingival disease*"  OR  "Gingival inflammation*"  OR  pericoronitis  OR  "gum bleed*"  OR  "dental bleed*"  OR  ( ( gum  OR  dental  OR  gingival  OR  gingiva )  W/3  ( haemorrhage  OR  hemorrhage  OR  bleed* ) ) ) ) )  AND  ( TITLE-ABS-KEY ( ( depress* ) ) )

Key: TITLE-ABS-KEY = TITLE = title, ABS = abstract, KEY = a combined field that searches author supplied keywords, EMTREE subject headings, other keywords, trade names and chemical names. W/n = word distance of maximum n words.

**Web of Science Core Collection: Citation Indexes**Science Citation Index Expanded (SCI-EXPANDED) --1900-present, Social Sciences Citation Index (SSCI) --1900-present, Arts & Humanities Citation Index (A&HCI) --1975-present, Conference Proceedings Citation Index- Science (CPCI-S) --1990-present, Conference Proceedings Citation Index- Social Science & Humanities (CPCI-SSH) --1990-present, Emerging Sources Citation Index (ESCI) --2015-present. Data last updated: 2020-11-26

Date of search: 2020-11-27
Retrieved references: 437

| # 3 | 437 | #2  AND  #1  Indexes=SCI-EXPANDED, SSCI, A&HCI, CPCI-S, CPCI-SSH, ESCI Timespan=All years |
| --- | --- | --- |
| # 2 | 649,156 | TS=(Depress*)  Indexes=SCI-EXPANDED, SSCI, A&HCI, CPCI-S, CPCI-SSH, ESCI Timespan=All years |
| # 1 | 52,937 | TS=  (gingivitis or periodontitis or "periodontal disease*" or "periodontal inflammation*" or peri-implantitis or "Gingival disease*" or "Gingival inflammation*" or pericoronitis or gum bleed* or dental bleed* or ((gum or dental or gingival or gingiva)  NEAR/3  (haemorrhage or hemorrhage or bleed*) ))  Indexes=SCI-EXPANDED, SSCI, A&HCI, CPCI-S, CPCI-SSH, ESCI Timespan=All years |

Key: TS = topic, which includes title, abstract, author keywords and Web of Science Keywords Plus. NEAR/n = word distance of maximum n words.

**Apendix 1.1 Search Update**

Update search for ‘‘Biomarkers common for inflammatory periodontal disease and depression: A systematic review and meta-analysis’’

2022-01-27

An update search was done on January 27^th^ 2022, by Marte Ødegaard, academic librarian at the Medical library, University of Oslo. The update search resulted in 514 references before duplicates were removed, and 207 references after.

**Embase, Ovid MEDLINE(R), APA PsycInfo**

Embase Classic+Embase <1947 to 2022 January 26>, Ovid MEDLINE(R) ALL <1946 to January 26, 2022>, APA PsycInfo <1806 to January Week 3 2022>

Date of search: 2022-01-27

Retrieved references: Embase 199, medline 57, PsycInfo 4.

| 1 | (gingivitis/ or gingivitis, necrotizing ulcerative/ or peri-implantitis/ or exp periapical periodontitis/ or periodontitis/ or aggressive periodontitis/ or chronic periodontitis/ or periapical periodontitis/ or periodontal abscess/ or periodontal pocket/ or Pericoronitis/ or Gingival Hemorrhage/) use medall | 43479 |
| --- | --- | --- |
| 2 | (gingiva disease/ or gingivitis/ or periodontitis/ or aggressive periodontitis/ or chronic periodontitis/ or periodontal disease/ or periimplantitis/ or gingiva disease/ or gingiva bleeding/) use emczd | 100786 |
| 3 | (gingivitis or periodontitis or periodontal disease* or periodontal inflammation* or peri-implantitis or Gingival disease* or Gingival inflammation* or pericoronitis or gum bleed* or dental bleed* or ((gum or dental or gingival or gingiva) adj3 (haemorrhage or hemorrhage or bleed*))).tw,kw,kf,hw,sh. | 195715 |
| 4 | 1 or 2 or 3 | 205124 |
| 5 | (Depression/ or exp Depressive Disorder/) use medall | 239274 |
| 6 | exp depression/ use emczd | 542277 |
| 7 | (Depression or depressive).tw,kf,kw. | 1362967 |
| 8 | (exp major depression/ or atypical depression/) use psyh | 144862 |
| 9 | 5 or 6 or 7 or 8 | 1602398 |
| 10 | 4 and 9 | 1382 |
| 11 | limit 10 to yr="2020 -Current"  Embase 199  Medline 57  PsycInfo 4 | 260 |

Key: /=medical subject heading (MeSH) in Medline, /=EMTREE subject headings in EMBASE, / = The *PsycINFO* Thesaurus, tw= text words (words in title, abstract, key concepts in PsycInfo), kw = author supplied keywords in Embase, kf = author supplied keywords in Medline, sh=Medical Subject Headings (MeSH), ADJn = word distance of maximum n words.

**Cochrane Library**

Date of search: 2022-01-27
Retrieved references: 7 in Trials

| #1 | ([mh gingivitis] or [mh "gingivitis, necrotizing ulcerative"] or [mh "peri-implantitis"] or [mh "periapical periodontitis"] or [mh periodontitis] or [mh "aggressive periodontitis"] or [mh "chronic periodontitis"] or [mh "periapical periodontitis"] or [mh "periodontal abscess"] or [mh "periodontal pocket"] or [mh Pericoronitis] or [mh "Gingival Hemorrhage"]) | 4686 |
| --- | --- | --- |
| #2 | (gingivitis or periodontitis or periodontal disease* or periodontal inflammation* or peri-implantitis or Gingival disease* or Gingival inflammation* or pericoronitis or gum bleed* or dental bleed* or ((gum or dental or gingival or gingiva) NEAR/3 (haemorrhage or hemorrhage or bleed*))):ti,ab,kw | 12677 |
| #3 | #1 OR #2 | 12932 |
| #4 | ([mh Depression] or [mh "Depressive Disorder"]) | 22657 |
| #5 | depress*:ti,ab,kw | 94761 |
| #6 | #4 or #5 | 94820 |
| #7 | #3 and #6 with Publication Year from 2020 to 2022, in Trials | 7 |

Key: ti = title, ab = abstract, kw = keyword, [mh XX] = medical subject heading (MeSH).

**Scopus (Elsevier, 1788-2020)**
Date of search: 2022-01-27
Retrieved references: 181

( TITLE-ABS-KEY ( ( gingivitis  OR  periodontitis  OR  "periodontal disease*"  OR  "periodontal inflammation*"  OR  peri-implantitis  OR  "Gingival disease*"  OR  "Gingival inflammation*"  OR  pericoronitis  OR  "gum bleed*"  OR  "dental bleed*"  OR  ( ( gum  OR  dental  OR  gingival  OR  gingiva )  W/3  ( haemorrhage  OR  hemorrhage  OR  bleed* ) ) ) ) )  AND  ( TITLE-ABS-KEY ( ( depress* ) ) )  A ND ( LIMIT-TO ( PUBYEAR , 2022 ) OR LIMIT-TO ( PUBYEAR , 2021 ) OR LIMIT-TO ( PUBYEAR , 2020 ) )

Key: TITLE-ABS-KEY = TITLE = title, ABS = abstract, KEY = a combined field that searches author supplied keywords, EMTREE subject headings, other keywords, trade names and chemical names. W/n = word distance of maximum n words.

**Web of Science Core Collection: Citation Indexes**Science Citation Index Expanded (SCI-EXPANDED) --1900-present, Social Sciences Citation Index (SSCI) --1900-present, Arts & Humanities Citation Index (A&HCI) --1975-present, Conference Proceedings Citation Index- Science (CPCI-S) --1990-present, Conference Proceedings Citation Index- Social Science & Humanities (CPCI-SSH) --1990-present, Emerging Sources Citation Index (ESCI) --2015-present. Data last updated: 2020-11-26

Date of search: 2020-01-27
Retrieved references: 66

|  |  |  |
| --- | --- | --- |
| # 3 | 66 | #2  AND  #1  Indexes=SCI-EXPANDED, SSCI, A&HCI, CPCI-S, CPCI-SSH, ESCI Timespan= 2020-11-01 - 2022-01-27 |
| # 2 | 715520 | TS=(Depress*)  Indexes=SCI-EXPANDED, SSCI, A&HCI, CPCI-S, CPCI-SSH, ESCI Timespan=All years |
| # 1 | 59 989 | TS=  (gingivitis or periodontitis or "periodontal disease*" or "periodontal inflammation*" or peri-implantitis or "Gingival disease*" or "Gingival inflammation*" or pericoronitis or gum bleed* or dental bleed* or ((gum or dental or gingival or gingiva)  NEAR/3  (haemorrhage or hemorrhage or bleed*) ))  Indexes=SCI-EXPANDED, SSCI, A&HCI, CPCI-S, CPCI-SSH, ESCI Timespan=All years |

Key: TS = topic, which includes title, abstract, author keywords and Web of Science Keywords Plus. NEAR/n = word distance of maximum n words.

| **Appendix 2. Key confounding variables measured and adjusted statistically for their impact on the relationship between IPD and depression in individual studies** | | |
| --- | --- | --- |
| **Study** | **Consideration for key confounding variables** | **Confounding factors and background variables** |
| Bawankar (2018) | no | smoking |
| Breivik (2015) | yes | age and sex matching, pups from the same rearing litter in each experimental group. Identical standard housing conditions, blinded radiographic examination of periodontal bone loss. A potential source of bias could also arise from non-blinding of observer in the behavioral testing. |
| Breivik (2006) | yes | weight, identical standard housing conditions, blinded radiographic examination of periodontal bone loss. A potential source of bias could also arise from non-blinding of observer in the behavioral testing. |
| Cakmak (2014) | yes | age, sex, occupation, education level, monthly income, smoking |
| Cakmak (2019) | yes | age, sex, education, employment, monthly income, smoking |
| Cakmak (2016) | yes | age, sex, education, employment, monthly income, smoking |
| Cohen-Cole (1983) | yes | age, sex, dental hygiene |
| da Silva (2015) | yes | anxiety, quality of life, oral hygiene |
| Fenol (2017) | no | not reported |
| Gomes (2018) | yes | age, sex, education, waist circumference, hypertension, smoking |
| Johannsen (2007) | yes | age, education, smoking, antidepressant use, marital status |
| Johannsen (2006) | yes | age, education, smoking, antidepressant use, marital status |
| Karimi (2017) | no | age, gender, education level |
| Katuri (2016) | no | smoking, brushing, alcoholism |
| Kurer (1995) | no | anxiety |
| Leira (2019) | yes | hypertension, Diabetes, Hypercholesterolemia, Bruxism, Depression, Smoking, Fibromyalgia, education level, stress, obesity |
| Martínez (2021) |  | blinding of depression-like behavior but does not say anything about periodontal status-assessment |
| Moss (1996) | yes | smoking, income, sex, age |
| Nascimento (2018) | yes | sex, income, smoking, BMI, maternal schooling level |
| Petit (2020) | yes | anxiety, stress, smoking, negative coping |
| Petit (2021) |  | Age, sex, smoking, alcohol use, marital status as well as anxiety, stress, coping skills |
| Rahate (2021) | yes | The effect of age and sex were investigated separately |
| Refulio (2013) | no | Not reported |
| Rodriguez Franco (2020) | yes | age, education, socioeconomic status, civil status, occupation |
| Rosania (2009) | yes | family history, gender, smoking, hygiene |
| Solis (2016) * | yes* | smoking, age, gender, race, marital status, income, BMI, employment |
| Wang (2019) | no | age matching, identical standard housing conditions. Limited information was available on how randomization was done. |
| Zhang (2021) | no | not adjusted for age or socioeconomic status |
|  |  |  |
| Note: * based on a previous study (Solis, A.C. et al., 2014. Evaluation of periodontitis in hospital outpatients with major depressive disorder. J. Periodontal Res. 49 (1), 77–84.) | | |

| **Study** | **Selection bias** | | | **Performance bias** | | **Detection bias** | | **Attrition bias** | **Reporting bias** | **Other** |
| --- | --- | --- | --- | --- | --- | --- | --- | --- | --- | --- |
|  | Sequence generation | Baseline characteristics | Allocation concealment | Random housing | Blinding | Random outcome assessment | Blinding | Incomplete outcome data | Selective outcome reporting | Other sources of bias |
|  | 1. Was the allocation sequence adequately generated and applied? | 2. Were the groups similar at baseline or were they adjusted for confounders in the analysis? | 3. Was the allocation adequately concealed? | 4. Were the animals randomly housed during the experiment? | 5. Were the caregivers and/or investigators blinded from knowledge which intervention each animal received during the experiment? | 6. Were animals selected at random for outcome assessment? | 7. Was the outcome assessor blinded? | 8. Were incomplete outcome data adequately addressed? | 9. Are reports of the study free of selective outcome reporting? | 10. Was the study apparently free of other problems that could result in high risk of bias? |
| Breivik (2015) | no | yes | unclear | no | no | no | unclear | unclear | yes | yes |
| Breivik (2006) | no | yes | unclear | no | unclear | no | yes | unclear | yes | yes |
| Martinez (2021) | yes | yes | unclear | no | unclear | no | yes | yes | yes | yes |
| Wang (2019) | no | yes | unclear | unclear | unclear | no | unclear | unclear | yes | no |
|  |  |  |  |  |  |  |  |  |  |  |

**Appendix 3a. Quality Assessment of Included Animals Studies by SYRCLE’s tool**

| **Appendix 3b. Quality Assessment of Included Observational Cohort and Cross-Sectional Studies on Humans by by NIH tool** | | | | | | | | | | | | | | |  | | |  |
| --- | --- | --- | --- | --- | --- | --- | --- | --- | --- | --- | --- | --- | --- | --- | --- | --- | --- | --- |
| *Study* | *1.Was the research question or objective in this paper clearly stated?* | *2. Was study population clearly specified and defined?* | *3. Was the participation rate of eligible persons at least 50%?* | *4. Were all subjects selected/recruited from same/similar populations? Inclusion/exclusion criteria being prespecified and applied uniformly to all participants?* | *5. Was a sample size justification, power description, or variance and effect estimates provided?* | *6. For the analyses in this paper, were the exposure of interest measured prior to the outcome(s) being measured?* | *7. Was timeframe sufficient so that one could reasonably expect exposure-outcome it existed?* | *8. For exposures that can vary in amount or level, did the study examine different levels of the exposure as related to the outcome (as categories or continuous)?* | *9. Were exposure measures clearly defined, valid, reliable, implemented consistently across all participants?* | *10. Was the exposure assessed more than once over time?* | *11. Were outcome measures clearly defined, valid, reliable, and implemented consistently across all study participants?* | *12. Were outcome assessors blinded to exposure status of participants?* | *13. Was loss to follow-up after baseline 20% or less?* | *14. Were key potential confounding variables measured and adjusted statistically for their impact on exposure-outcome relationship?* | | *Score* | *Study Quality* | |
| Cakmak (2014) | 1 | 1 | 1 | 1 | 1 | 0 | NR | 1 | 1 | 0 | 1 | 0 | 1 | 0 | | 8 | fair | |
| Cakmak (2019) | 1 | 1 | NR | 1 | 1 | 0 | NR | 1 | 1 | 0 | 1 | 0 | 1 | 0 | | 10 | good | |
| Cakmak (2016) | 1 | 1 | 1 | 1 | 1 | 1 | 1 | 0 | 1 | 0 | 1 | NR | 1 | 0 | | 10 | good | |
| da Silva (2015) | 1 | 0 | 1 | 1 | 1 | 1 | NR | 1 | 0 | 0 | 1 | NR |  | 1 | | 9 | fair | |
| Fenol (2017) | 1 | 1 | 1 | 1 | 1 | 0 | 0 | 1 | 0 | 0 | 1 | 1 | NR | 0 | | 7 | fair | |
| Gomes (2018) | 1 | 0 | 1 | 1 | 0 | 1 | NR | 1 | 1 | 0 | 1 | 1 | 1 | 1 | | 9 | fair | |
| Katuri (2016) | 1 | 0 | 0 | 1 | 0 |  | 0 | 0 | 1 | 0 | 1 | 0 | 0 | 0 | | 4 | poor | |
| Kurer (1995) | 1 | 0 | 0 | 1 | 0 | 1 | 1 | 0 | 1 | 1 | 1 | 0 | 1 | 0 | | 8 | fair | |
| Nacimento(2018) | 1 | 1 | 0 | 1 | 0 | 1 | 0 | 1 | 1 | 0 | 1 | 0 | 0 | 1 | | 8 | fair | |
| Petit (2020) | 1 | 1 | 0 | 1 | 1 | 1 | 1 | 0 | 1 | 1 | 1 | 1 | 0 | 1 | | 11 | good | |
| Petit (2021) | 1 | 1 | 1 | 1 | 0 | 1 | 1 | 1 | 1 | 1 | 1 | 1 | 1 | 1 | | 13 | good | |
| Rahate (2021) | 1 | 1 | 1 | 1 | 1 | NR | NR | NR | 1 | NR | 1 | 1 | NR | 1 | | 9 | fair | |
| Refulio (2013) | 1 | 1 | 0 | 1 | 0 | 0 | 0 | 0 | 1 | 0 | 1 | 0 | 0 | 0 | | 5 | poor | |
| Rodriguez(2020) | 0 | 1 | 0 | 0 | 1 | 0 | 0 | 0 | 1 | 1 | 1 | 0 | 1 | 1 | | 7 | fair | |
| Rosania (2009) | 1 | 1 | 0 | 1 | 0 | 0 | 0 | 0 | 1 | 0 | 1 | 0 | 0 | 1 | | 6 | fair | |
| Solis (20216) | 1 | 0 | 1 | 1 | 0 | 1 | 0 | 1 | 1 | 0 | 1 | 0 | 1 | 1 | | 9 | good | |
| Zhang (2021) | 1 | 1 | 1 | 0 | 1 | NA | NA | NR | 1 | NR | 1 | 1 | 1 | 0 | | 8 | fair | |

Note to Appendix 3b: Responses corresponding to NR (not reported), CD (cannot determine) or NA (not applicable) were coded as 0 for totalling the consensus score. Study quality reported as 'good', 'fair' and 'poor' were based on overall evaluations of possible sources of bias in each study.

**Appendix 3c. Quality Assessment of Included Case-Control Studies on Humans by NIH tool**

| *Study* | *1. Was the research question or objective in this paper clearly stated and appropriate?* | *2. Was the study population clearly specified and defined?* | *3. Did the authors include a sample size justification?* | *4. Were controls selected or recruited from the same or similar population that gave rise to the cases (including the same timeframe)?* | *5. Were the definitions, inclusion and exclusion criteria, algorithms or processes used to identify or select cases and controls valid, reliable, and implemented consistently across all study participants?* | *6. Were the cases clearly defined and differentiated from controls?* | *7. If less than 100 percent of eligible cases and/or controls were selected for the study, were the cases and/or controls randomly selected from those eligible?* | *8. Was there use of concurrent controls?* | *9. Were the investigators able to confirm that the exposure/risk occurred prior to the development of the condition or event that defined a participant as a case?* | *10. Were the measures of exposure/risk clearly defined, valid, reliable, and implemented consistently (including the same time period) across all study participants?* | *11. Were the assessors of exposure/risk blinded to the case or control status of participants?* | *12. Were key potential confounding variables measured and adjusted statistically in the analyses? If matching was used, did the investigators account for matching during study analysis?* | *Score* | *Overall Study Quality* |
| --- | --- | --- | --- | --- | --- | --- | --- | --- | --- | --- | --- | --- | --- | --- |
| Bawankar (2018) | 1 | 0 | 1 | 1 | 1 | 0 | 0 | 0 | 1 | 1 | 0 | 0 | 6 | fair |
| Cohen-Cole (1983) | 1 | 1 | 0 | 1 | 0 | 0 | 0 | 0 | 1 | 1 | 0 | 1 | 6 | fair |
| Johannsen (2007) | 1 | 1 | 0 | 1 | 0 | 1 | 1 | 1 | 1 | 0 | NR | 0 | 7 | good |
| Johannsen (2006) | 1 | 1 | 0 | 1 | 0 | 1 | 1 | 1 | 0 | 0 | 0 | 1 | 7 | good |
| Karimi (2017) | 1 | 0 | 0 | 1 | 0 | NR | NR | 1 | 0 | 0 | 0 | 0 | 3 | poor |
| Leira (2019) | 1 | 1 | 0 | 1 | 1 | 1 | NR | 1 | 0 | 1 | 0 | 1 | 8 | good |
| Moss (1996) | 1 | 1 | 0 | 1 | 1 | 1 | 0 | 1 | 0 | 0 | 0 | 1 | 7 | good |
| Note: Values with NR represent data not reported or unclear. Overall study quality is a reflection of the total evaluation of potential biases inherent in each study. | | | | | | | | | | | | | | |
